# The body in isolation: The physical health impacts of incarceration in solitary confinement

**DOI:** 10.1371/journal.pone.0238510

**Published:** 2020-10-09

**Authors:** Justin D. Strong, Keramet Reiter, Gabriela Gonzalez, Rebecca Tublitz, Dallas Augustine, Melissa Barragan, Kelsie Chesnut, Pasha Dashtgard, Natalie Pifer, Thomas R. Blair

**Affiliations:** 1 Department of Criminology, Law and Society, University of California, Irvine, Irvine, California, United States of America; 2 Department of Psychological Sciences, University of California, Irvine, Irvine, California, United States of America; 3 Department of Criminology and Criminal Justice, The University of Rhode Island, Kingston, Rhode Island, United States of America; 4 Department of Psychiatry, Southern California Permanente Medical Group, Downey, Los Angeles, California, United States of America; University of North Carolina at Chapel Hill, UNITED STATES

## Abstract

We examine how solitary confinement correlates with self-reported adverse physical health outcomes, and how such outcomes extend the understanding of the health disparities associated with incarceration. Using a mixed methods approach, we find that solitary confinement is associated not just with mental, but also with physical health problems. Given the disproportionate use of solitary among incarcerated people of color, these symptoms are most likely to affect those populations. Drawing from a random sample of prisoners (n = 106) in long-term solitary confinement in the Washington State Department of Corrections in 2017, we conducted semi-structured, in-depth interviews; Brief Psychiatric Rating Scale (BPRS) assessments; and systematic reviews of medical and disciplinary files for these subjects. We also conducted a paper survey of the entire long-term solitary confinement population (n = 225 respondents) and analyzed administrative data for the entire population of prisoners in the state in 2017 (n = 17,943). Results reflect qualitative content and descriptive statistical analysis. BPRS scores reflect clinically significant somatic concerns in 15% of sample. Objective specification of medical conditions is generally elusive, but that, itself, is a highly informative finding. Using subjective reports, we specify and analyze a range of physical symptoms experienced in solitary confinement: (1) skin irritations and weight fluctuation associated with the restrictive conditions of solitary confinement; (2) un-treated and mis-treated chronic conditions associated with the restrictive policies of solitary confinement; (3) musculoskeletal pain exacerbated by both restrictive conditions and policies. Administrative data analyses reveal disproportionate rates of racial/ethnic minorities in solitary confinement. This analysis raises the stakes for future studies to evaluate comparative prevalence of objective medical diagnoses and potential causal mechanisms for the physical symptoms specified here, and for understanding differential use of solitary confinement and its medically harmful sequelae.

## Introduction

The health implications of solitary confinement have received increasing attention in recent years [1, 2]. Although both the conditions and terms defining solitary confinement are contested, the practice generally involves being locked in a cell alone, for 22 or more hours per day, with extremely limited access to human contact and communication [3, 4]. Until recently, however, research on the health consequences of solitary confinement has focused almost entirely on the negative impacts on mental health [4–8]. While initial studies focused on the effects of sensory deprivation [9–11], recent work has examined the impacts of social deprivations [12, 13]. Such studies have found that placement in solitary confinement has been associated with symptoms of increased psychological distress, such as anxiety, depression, paranoia, and aggression [14–16]. A 2018 study, for instance, found that prisoners who had spent time in solitary confinement were three times as likely to exhibit symptoms of post-traumatic stress disorder (PTSD) than those who had not [17]. Some researchers, however, have argued that the psychological harms of solitary confinement are limited or unverified [18, 19]. The analyses on which such opinions rely have, in turn, been criticized for neglecting existing literature and for other serious methodological concerns, including an inability to isolate exposure to solitary confinement, lack of specificity about variability and comparability in actual conditions of confinement, and the inapplicability of psychological assessment scales in the prison context [1, 20].

In a study examining the lived experiences of solitary confinement in Washington state, we, too, focused on documenting the mental health impacts of the practice, through qualitative interviews with a random sample of 106 prisoners in long-term solitary confinement, application of a Brief Psychiatric Rating Scale (BPRS) assessment at two points in time with those prisoners, review of medical health records, and analysis of administrative data. To our surprise, however, we found that, after anxiety and depression, the third most common significant health symptoms experienced by our subjects were “somatic concerns,” defined by the BPRS as “concerns over present bodily health” [21]. This observation led us to examine our data systematically for evidence of the impacts of solitary confinement on physical health, and to consider the implications of such impacts for understanding the health disparities enacted by solitary confinement, and by incarceration more broadly.

Existing research on the physical health impacts of incarceration demonstrates the need for further study of both the medical effects of isolation and its racially disparate impacts, especially considering that there are roughly 80,000 people in isolation units nationwide, and this population includes a disproportionate number of racial minorities relative to the overall prison population [22]. Outside of prison, health disparities by race and ethnicity are well attested by existing epidemiologic research [23]. Notably, Black and other racial/ethnic minorities consistently show lower life expectancies and worse mental health outcomes than whites [24–27]. Health disparities persist, and are magnified, among the incarcerated population, where people of color are disproportionately represented [28–30]. In particular, people in prison are at higher risk than the general population for substance use disorders, psychiatric disorders, victimization, and chronic infectious diseases such as HIV and hepatitis C [31–34]. Incarceration has also been shown to exacerbate chronic illnesses such as obesity [35], hypertension, and asthma [36, 37, 29], and formerly incarcerated people experience disparately adverse health outcomes more generally [38]. The interaction between the disparate impacts of race and incarceration on health mean that mass incarceration itself has been identified as a social determinant of health for Black men in the United States [39, 40].

Solitary confinement amplifies the disproportionately adverse effects of mass incarceration on people of color. Depending on the composition of the prison system, Blacks and/or Latinos are often over-represented in solitary confinement relative to their (over)representation in the general prison population [40–44]. Any concentrated health disadvantages affecting people in prison, and especially people of color, is potentially even more concentrated among those living in solitary confinement. Moreover, existing evidence suggests that conditions of solitary confinement exacerbate health problems and pose a significant public health risk [45, 42].

Studies reporting the physical health impacts of solitary confinement have tended to focus on issues like self-harm and suicide [46, 47, 8]. One recent study has examined the cardiovascular health burdens of solitary confinement [45]. A growing body of neuroscience literature has examined the effects of solitary confinement on the brains of lab animals, documenting that lab animals in isolated environments have “a decrease in the anatomical complexity of the brain” compared to those in more enriched environments [48, 49] (p70). One recent study found similar effects in Antarctic expeditioners: a shrinking hippocampus, hypothesized to be a result of the isolated and monotonous environment [50]. Such neuroscience research has been used in litigation to argue that there is likely a similar effect on humans imprisoned in solitary confinement [51, 48, 49]. The associations between solitary confinement, self-harm, and lab animals’ brain structure suggest comorbidity between mental health and physical injury in solitary confinement [1, 48].

The physical effects of solitary confinement manifest well beyond release from isolation, and from incarceration overall. One recent study has examined post-release mortality (from all causes, including suicide, murder, and drug overdose) associated with previous time in solitary confinement: people who had spent time in solitary confinement in North Carolina between 2000 and 2015 were 24% more likely to die in their first year after release than former prisoners who had not spent time in solitary confinement [52]. Similarly, a 2020 study found that Danish people who had spent time in solitary confinement had higher mortality within five years of being released from prison compared to those who never spent time in solitary confinement [53]. This mortality risk associated with solitary confinement exceeds the already high mortality risk associated with incarceration and release from prison [52–54].

In sum, while many studies have examined the relationship between incarceration and health, and some studies have examined the relationship between solitary confinement and mental health, the existing literature lacks analysis of disparate physical health outcomes across levels and severity of confinement [2], especially within isolation, and for incarcerated people of color. To our knowledge, this article is the first of its kind to consider associations between solitary confinement and a range of physical health problems, and to incorporate explicit consideration of racial health disparities.

## Methods and materials

To explore the physical health problems experienced in isolation, we draw upon a research study of people in long-term solitary confinement in the Washington State Department of Corrections (WADOC). The study consists of four dimensions of participant data: 1. surveys of prisoners in solitary confinement; 2. in-depth interviews with a random sample of prisoners in solitary confinement; 3. reviews of the medical (covering mental and physical health) files, as well as the disciplinary records, for this subset of prisoners; and 4. administrative data for the entire 2017 prison population provided by the WADOC. Data was collected in 2017 and 2018.

### Setting

WADOC is a mid-sized state prison system, with the 12^th^ lowest rate of incarceration of the 50 United States [20]. The state and its prison system have a reputation for being progressive, including engaging in reforms to minimize the use of solitary confinement statewide, and for inviting independent academic researchers to evaluate conditions and programs [20, 55–57]. Five of the state’s 12 prison facilities have an Intensive Management Unit (IMU), an all-male unit or building, housing people in solitary confinement (with highly restricted access to commissary, phones, radios, televisions, visitors, and roughly 10 hours per week out-of-cell) for durations ranging from months to years. Our study focused on people within the IMUs on “maximum custody status”: the highest security level assigned to state prisoners housed in the IMU for an indeterminate period, usually following one or more rule violations, with return to the general prison population contingent on meeting specific benchmarks.

### Participant sampling

First, paper surveys were distributed in-person (and collected on the same day) to all 363 people on maximum custody status in the five state IMUs in the spring of 2017. Next, during the summer of 2017, roughly one-third (29%) of all 363 people on maximum custody status in IMUs were interviewed, selected from randomly ordered lists of the population of each IMU. One year later (2018), all participants from our initial random sample, who were still incarcerated one year later, including those no longer housed in the IMU, were re-interviewed. We also reviewed paper medical and disciplinary files for each consenting, year-one interview participant. Interviews, file reviews, and observations were conducted over two separate three-week periods in the summers of 2017 and 2018, by a total of 13 research team members. Finally, we received administrative data on all people within the state prison system as of July 1, 2017.

### Research team training

All interviewers underwent an extensive training process, including more than 20 hours of meetings to learn about conditions in Washington IMUs and develop the interview instrument. Interviewers completed an additional 20 hours of a standardized training protocol for administering the BPRS in clinical settings: 16 hours of in-person symptom assessment training sessions with a leading expert in BPRS research—Dr. Joe Ventura—in year one, and four hours of refresher training prior to the year-two interviews. Dr. Ventura conducted an interrater reliability analysis confirming trained raters met the minimum standard of an ICC = .80 or greater for the BPRS. This extensive training sought to ensure that the 13 team members (9 women and 4 men; 9 white and 4 non-white), all faculty (4) or doctoral students (9) with expertise in prisons and prior interview experience in secure confinement settings, identified and addressed any pre-existing assumptions about the population being studied and minimized any possible bias as a result of inconsistent interpretation or application of questions and assessments. Eight of the authors on this paper participated in interviews; two participated only in data analysis.

### Interviews

On site in the Washington State IMUs, after the random sample was drawn and willing participants identified, prison staff escorted participants, one at a time, to a confidential area (monitored visually but *not* aurally by WADOC staff). Prior to conducting interviews, interviewers informed participants that participation was voluntary and would not involve incentives, administrative or otherwise; that refusal would not affect them adversely; and that all information shared would be protected and anonymized, unless it pertained to “an imminent security-related threat.” (In the highly restrictive setting of the IMU, any incentive beyond providing human contact and an attentive listener would both run the risk of being an undue influence, coercing participation, and be administratively prohibited.) Participants provided oral consent to participate in the interview. Immediately following interviews, interviewers asked participants whether they consented to the research team reviewing their medical files and to participating in one-year follow-up interviews. All participants agreed orally to re-interviews, and all but two (n = 104) consented in writing to medical file reviews. Following interviews, interviewers reviewed consenting participants’ paper medical files for histories of diagnoses, prescriptions, and substance abuse status; WADOC additionally provided electronic administrative health and disciplinary files for all 104 consenting participants, as well as comparable, population-level data for all people incarcerated in the system in July 2017.

All identifiable data collected for this research, including interview audio recordings, transcripts, BPRS score sheets, medical file notes, and administrative data, was stored either in a locked filing cabinet in a locked office of the university or in a secure server space, accessible only through multi-factor identification to a subset of study team members participating in data cleaning and linking. The University of California, Irvine, Office of Research Institutional Review Board approved this study (HS 2016–2816), and the WADOC Research Department reviewed this approval.

### Data collection instruments

The initial paper survey of people confined in the WADOC IMU consisted of 36 numbered questions (each containing a combination of yes/no, ordinal bubble options, and short answer sub-questions leaving participants an opportunity to explain or elaborate on their answers) about experiences in IMUs, conditions of confinement, health and well-being, and demographic background, drawing from existing studies on prisons and prisoner experiences [58–62]. Survey in S1 Text. In all, there were 89 substantive items on the survey (excluding demographic questions) coded quantitatively as cardinal (e.g., number of days in IMU), ordinal (e.g., daily, weekly, monthly describing frequency of interactions), or categorical (e.g., yes/no) variables. In this paper, we report on the results of a sub-set of five quantitatively coded items relating to health from this larger survey. This survey functioned as a pilot instrument for the in-person interviews, allowing us to ensure questions were clear and relevant, yielding responses comparable across subjects and institutional contexts, and providing our interviewers with a baseline description of participants’ experiences prior to conducting qualitative interviews.

The qualitative interview instrument consisted of 96 numbered semi-structured questions (each containing a combination of yes/no questions and probing, open-ended follow-up questions) seeking elaboration on responses from the survey questions and also drawing from existing studies on prisons and prisoner experiences [60–63], including conditions of daily life (prior to and during isolation), perceived state of physical and mental health, access to medical treatment, and experiences with required programming in the IMU, among other topics. Interview instrument in S2 Text. We first used the instrument at the smallest IMU in Washington, interviewing 15 prisoners, and we then revised both the wording and ordering of questions for maximum clarity and engagement in the remaining 91 interviews we conducted across the four other IMUs in the state. In total, 40 of the substantive items on the interview instrument (excluding 10 demographic questions and 18 embedded questions designed to establish BPRS scores and/or assess orientation) were coded quantitatively as cardinal (e.g., How much does it cost to see a doctor or dentist?) or categorical (e.g., Have you noticed any changes in your health since you have been in this IMU?) variables. Such questions always included open-ended follow-up questions (e.g., Can you describe those changes?). Transcribed responses to those open-ended follow-up questions, which related in any way to physical health, constitute the central source of data analyzed in this paper.

Interviews ranged in length from 45 minutes to three hours. Follow-up interviews lasted between 45 minutes and two hours. The condensed year-two instrument contained approximately 70 questions, largely replicating the year-one questions, but excluding the background demographic questions and questions about experiences over time in prison, and adjusting some questions to address prisoners’ current (and often different) housing status.

As part of both initial and follow-up instruments, interviewers administered the BPRS psychological assessment both during (for the 14 self-report questions) and immediately following (for the 10 observational items regarding a participant’s demeanor, engagement, and speech) the interviews. For self-report questions (14 items), embedded in the interview guide, interviewers asked about the presence of symptoms in the two weeks prior, per BPRS standard [20].

Interviews were assigned a randomly generated identifier, audio recorded (with permission), professionally transcribed in Microsoft Word, translated (in one case, from Spanish into English) by research team members, systematically stripped of identifying information, and then systematically checked against the original audio by the original interviewer(s). Interviews were linked, by random identifier to BPRS score sheets (which were scanned and entered into Microsoft Excel for descriptive statistical analysis), scanned medical file review notes, and WADOC administrative data.

### Data analysis & reporting

BPRS and other administrative data were imported into Statistical Package for Social Science (SPSS) (IBM, Armonk, NY) and Stata (StataCorp LLC, College Station, TX) to generate descriptive statistics, including the comparative prevalence of significant ratings on BPRS items and factors relating to physical health and demographics of the sample interview population as compared to: the IMU population, the overall state prison population, and the overall population of the state itself. Fisher’s exact test and McNemar’s test were performed to evaluate the relationships between BPRS ratings across housing location, time, and race/ethnicity; chi square tests of homogeneity were performed to compare racial/ethnic distributions in the IMU population, the general prison population, and the Washington state population. The demographic data utilizes a confidential data file from the WADOC.

Transcribed interviews were analyzed using Atlas-ti (ATLAS.ti Scientific Software Development GmbH, Berlin, Germany). Six team members, who had also conducted interviews, engaged in an iterative and recursive coding process. Consistent with the tenets of constructivist grounded theory, coders inductively explored how participants make meaning of their experiences (here: their time in solitary confinement) [63, 64]. This process included initial, line-by-line open-coding of a subset of transcripts, which generated a list of 214 codes, grouped into 11 major categories (e.g., Health) with sub-themes (e.g., physical health) [63]. Some of these initial codes and categories corresponded with specific questions on our interview instrument (most relevant for the instant analysis: question 29 concerned medical “kites,” and questions 30, 31, and 38 concerned physical health and somatic concerns). However, open-ended questions also yielded responses related to these topics and were so coded. Given the constraints of the prison setting (in-person contact is expensive and time-consuming; mail contact is not confidential because of prison censoring policies), participants have not provided systematic feedback on their transcripts or our findings. However, the year-two interviews did give research team members an opportunity to discuss year-one themes with participants.

All quotations presented in this paper were initially identified in the first phase of our coding process by one of three (out of our initial 214) codes: “somatic concerns,” “physical health,” or “kites” (the standard, slang term for a paper form handed to a correctional officer to request medical attention). Two coders then used intermediate focused coding techniques to

re-code these 319 quotes, exploring the relationship between solitary confinement conditions and policies and physical health problems, “transform[ing] basic data into more abstract concepts and allowing the theory to emerge from the data” [64 p. 5].

Notes from reviewing participants’ paper medical files corroborate details from the qualitative analysis that systematically anchors this data. Each participant has been assigned a pseudonym and, because we are also exploring the racially disparate impact of the health problems we identify, we specify each quoted participant’s self-identified race or ethnicity. We linked quotations to specific racial/ethnic identities only after quotations were selected for inclusion in this manuscript, as representative of the themes we identified in coding.

## Results

In total, 225 prisoners in IMU (62%), responded to our in-person survey. The refusal rate of initial interviews was 39% (67 out of 173 approached), comparable to similar studies of prisoners [15, 58, 59, 65]. The drop-out rate of our sample for the one-year follow-up interviews was comparable to other studies at 25%: there were 4 refusals; 21 institutional, out-of-state, and parole transfers precluding follow-up; and one death [58–61]. Our random sample of 106 (all-male) IMU prisoners reflects a mean age of 35; mean stay of 14.5 months in IMU; mean of 5 prior convictions resulting in prison sentences. Among our participants 42% were white; 12% were African American; 23% were Latino; 23% were “Other.” There were no significant differences between our participants and all people held in IMU at the time of our sample. People in the general prison population at the time of our sample are notably different as they are older, less violent in terms of criminal history, serving shorter sentences, less likely to be gang-affiliated, and less likely to be Latino than those held in IMU [20]. (We discuss racial differences across these populations further in the final results sub-section.)

### Prevalence of somatic concerns

As an initial basis for describing physical symptoms experienced in solitary confinement, we present a quantitative analysis of the prevalence of somatic concerns in our random sample of 106 people held in IMU, and the variability of these concerns across time and housing location. In 2017, 15% of participants reported having clinically significant (formally defined as a severity of 4 or higher out of a possible 7) somatic concerns (formally defined as “concern over present bodily health”) on the BPRS assessment [21]. In the 2018 re-interview sample, of the 80 respondents re-interviewed in the second year of the study, 12.5% reported clinically significant ratings of somatic concern.

While ratings of clinically significant somatic concern mostly varied within participants over time, our analysis indicated some persistence of somatic issues across the two assessment periods. Of those who reported clinically significant somatic concern in 2017 and who were re-interviewed in 2018 (12 respondents; 4 were unavailable for re-interview), 25% (3 respondents) indicated a persistence of clinically significant somatic issues in 2018. An exact McNemar’s test revealed no statistically significant relationship between the proportion of respondents reporting clinically significant somatic concerns in 2017 and 2018 (*p = 0*.*80)*.

In the initial 2017 assessment, all study subjects were housed in IMU. At the time of re-interview in 2018, 52 respondents had moved into the general prison population, while 28 remained in IMU. Of those who were still in IMU in 2018, 21% (6 of 28) reported clinically significant somatic concerns, compared to just 8% of those housed in the general prison population (4 of 52). While the descriptive data appear to demonstrate higher proportions of somatic concern in IMU settings, the difference was not statistically significant at the 95% confidence level (*p* = 0.09; Fisher’s exact test). No significant differences were observed in the distribution of clinically significant somatic concern ratings across racial and ethnic groups.

Complementing the BPRS assessment data from the random sample of 106 individuals in IMU custody, survey data collected from the full IMU population in 2017 further indicated the prevalence of somatic concerns among this population. Of the 225 survey respondents, 63% expressed health concerns; 48% were taking medication; 17% had arthritis; and 8% had experienced a fall in solitary confinement. Importantly for the analysis of emerging symptoms in particular, 82% replied “yes” to the question “Have you experienced any changes in yourself?” while in the IMU. These survey results, like the BPRS somatic concern results, benefit from triangulation with our qualitative data.

### Specifying physical symptoms

We identify three categories of physical symptoms people experience in solitary confinement, each associated with different aspects of IMU housing: symptoms associated with deprivation conditions, symptoms associated with deprivation policies limiting access to healthcare, and chronic musculoskeletal pain exacerbated by the intersection of deprivation conditions and deprivation policies. In each category, we analyze how the institution of solitary confinement shapes both physical health outcomes and perceptions of health for people housed in solitary confinement, revealing both the mechanisms of physical health deterioration and the accentuated comorbidity of physical and mental health in solitary confinement.

#### Deprivation conditions

Our participants described a range of physical ailments directly connected to the conditions of their confinement, especially the various deprivations of movement, provisions (from food to toiletries), and human contact inherent in the institutional restrictions defining solitary confinement. Skin irritations and weight fluctuations were the most common of these; participants experienced both as co-morbid with anxiety and other health issues.

Participants described rashes, dry and flaky skin, and fungus developing in isolation. They understood these conditions as being directly associated the poor air and water quality, irritating hygiene products, and lack of sun exposure inherent to their conditions of solitary confinement. People in the IMU (unlike those in the general prison population) usually cannot purchase or trade for alternative, higher-quality hygiene products; their cells have limited natural light (at best, a window far above eye-level; at worst, no window); and even the exercise areas frequently have limited natural light. Indeed, research has documented how isolation can cause vitamin D deficiency due to lack of natural light exposure [66].

As Joseph (white) explained, an ostensibly trivial physical problem, like dandruff, can inspire a sense of helplessness in the IMU:

Well I try not to [think about] what happens to my body…Because you’re going to obsess on it probably…Minor things become huge when you’re in segregation, and so, something that you–you as being free in society can alleviate by going to, you know, to [the store] or whatever, and just get a dandruff shampoo. You can’t do that here. And kiting medical and telling them “Hey, I have a severe problem with dermatitis, and my head’s itching and I’ve got bleeding scabs on my head,” or whatever the case may be, there’s nothing that we can do here. You’re SOL [shit out of luck].

Joseph’s inability to treat his skin irritations himself led to both helplessness and obsessiveness, further exacerbating the discomfort and potential health consequences of the issue. This case illustrates how a free person’s flaky skin or minor embarrassment becomes a potentially severe medical problem in solitary confinement, entailing bleeding scabs on the scalp.

Participants frequently experienced fluctuations in body weight and, as with skin irritations, connected these symptoms to conditions inherent to solitary confinement. What started as simple observations about diet, exercise, and appearance often turned into analyses of the impact of conditions of confinement on physical, as well as mental health. Simon (Black) discussed being “real worried” about his weight:

The only reason I know they’re not really giving us the calorie needs they’re supposed to give us, is because I feel like I’m losing more muscle than I am fat. And to lose more muscle than fat is because you’re not getting the nutrients that you need.

Not only is weight loss a significant source of anxiety for Simon, but he connects the deprivations of confinement–the lack of nutritious food and sufficient calories–to physical changes in his body. Whether his explanation is correct, or simple lack of physical activity is more likely to explain the changes accurately, IMU confinement ostensibly produced the change.

Participants also described restricting their own dietary intake, beyond the already limited rations (usually calculated to meet the minimum daily calorie intake standards), for a variety of reasons, from the quality of the food to their emotional state. Michael (Latino) described being suspicious of staff having tampered with his food: “I got my breakfast bowl and there was a tear on the plastic. […] Sometimes your mind plays tricks on you, like they’re trying to poison you or something.” While Michael noted that his suspicions were likely just in his mind, Philip (Black) asserted: “They was poisoning my food–they control everything. They can even manipulate the water. I’m so fucking serious; this place is highly technologically advanced.” For those like Michael and Philip, psychological states associated with the conditions of confinement (e.g., suspiciousness, paranoia, and potentially psychosis) caused them to restrict their food intake, resulting in weight loss. Indeed, both Michael and Philip had documented diagnoses of mental illness in their medical files; bipolar disorder and undifferentiated schizophrenia respectively. Food restrictions can, of course, lead to more imminently dangerous conditions, such as dehydration, electrolyte imbalances, or renal failure–none of which are likely to be subject to objective evaluation in the IMU, as we discuss further in the next sub-section on the impacts of deprivation policies.

Some prisoners made a more direct connection between their mental health, their dietary intake, and their physical health. For instance, Kai (Native American), said:

I don’t work out because I have a problem breathing …This is the first time I’ve ever done a program [IMU term] where I’ve felt like I was breaking. Because before I’d be working out… Now, I’m stuck in this …I’m battling mentally with everything going on. Which affected my body, effects my eating sometimes. I’ll just take the [food] tray but I’ll flush the stuff down the toilet.

As Kai suggests, in the IMU, exercise functions not only as a means to practice physical fitness, but also to provide structure for people to manage both their days and the mental strain of being in isolation. When asked a general question, like “how are you doing in the IMU?” many participants, like Kai, referenced whether or not they were engaging in exercise as a way to gauge how they were faring overall. People like Kai shared feelings of lethargy, or feeling too overwhelmed to do anything but lie around all day, induced by long periods in solitary confinement. Their weight fluctuated during these cycles: going down with regular and social exercise routines, going up with exercise-induced injuries or periods of lethargy. Concerns around exercise, diet, and the associated body weight fluctuations, like concerns with skin irritations, highlight the interdependence of physical and mental wellbeing for prisoners in the IMU.

#### Deprivation policies

Our participants described multiple situations in which official IMU policies and unofficial IMU practices exacerbated their physical ailments, especially their chronic health problems. Such policies and practices included the prioritization of security over care in emergency situations, disruptions in care upon transfer into the IMU, and overwhelming administrative hurdles to accessing care in the first place. If prisons are largely unequipped to provide the appropriate care and environment for chronic medical problems [67, 31], our findings reveal both the specific mechanisms by which solitary confinement policies amplify the usual bureaucratic challenges of accessing healthcare in prison and the kinds of physical health problems that go unaddressed as a result.

First, in cases of medical emergencies, people housed in the IMU have response buttons in their cells they can press to alert staff. However, many of the people we interviewed both doubted whether staff would respond swiftly enough in an actual emergency and worried about being punished with additional time in the IMU for activating an emergency response, if medical staff ultimately deemed their problem non-emergent. Indeed, prisoners perceived IMU policies as systematically prioritizing incapacitation over medical attention. Carl (white) described an incident where he experienced delayed care and was pepper sprayed after having suffered from a seizure, all because he was unable to comply with orders to stand following the episode:

I had a serious seizure. And I was laying on the floor, and I had defecated. I was laying in a puddle of puke…Well, [the guards] had come to the door, and I guess they had called medical…and they were standing there for 45 minutes yelling, “Stand up and cuff up so we can give you medical attention.” They did not pop the door and go in there and give me medical attention. And so, unknown to me, they popped the cuff port, and they sprayed OC [pepper spray] in there. And then they came in. They noticed that I was unconscious, and finally a nurse looked at my medical file and she’s, like, “he’s epileptic.”

In the tense environment of the IMU, where staff manage people with histories of violating prison rules, assaulting staff, and, often, serious mental health needs, immediate security concerns readily take priority over assessing medical histories and providing healthcare.

Second, simply being transferred into the IMU often disrupted care in dangerous ways. For instance, Julian (Hawaiian) described how, when he was transferred into a new solitary confinement unit, he had to restart the process of seeking treatment for (and even simple acknowledgement of) recurring kidney stones. Whereas he had fought and been able to receive x-rays and medication to help manage his kidney pain at his prior institution, he now found this fight to be futile at his new facility: “They’re just going to take me out of room, take me over there to medical, and they’re going to be like, oh here’s the hot water or hot bag or whatever.” And Tony (Native American/white) described a battery of physical and mental health issues–an enlarged prostate, a painful cyst that needed to be surgically removed, varicose veins, “chronic suicidal thoughts,” anxiety, and depression–all requiring medications, which he had difficulty maintaining access to in the IMU. For instance, he described how both his Amitriptyline, which partly treated his periodic limb movement sleep disorder, and his seizure medication, Dilantin, were both discontinued in the IMU, resulting in serious injuries to his foot and head.

Third, a number of bureaucratic hurdles and barriers discouraged people in the IMU from attempting to access healthcare at all, even in potentially life-threatening situations. In order to see a medical professional, people isolated in the IMU must fill out a paper request (a “kite”) and hand it to a correctional officer passing by, or report a concern to a nurse, who makes daily rounds passing by each cell in the IMU. The medical response happens either “cellfront,” with the person talking to the medical professional through his cell door, in earshot of others held in solitary confinement, or “by escort,” with the person in handcuffs and leg-cuffs, if not also belly chains and a hood, usually accompanied by at least two to four correctional officers, to a medical treatment area. Vitamins and over-the-counter medications like Tylenol, or as-needed medications like asthma inhalers, are kept outside of the cell and available only at specified times, or, again, by paper kite request. Throughout WADOC, people must pay $4 for non-emergency medical care (unless they are indigent, in which case WADOC provides care without a co-pay), but people held in the IMU have more restrictive caps on their overall spending for any needs, including healthcare, food, and toiletries, proportionally raising the relative cost of seeking care for non-emergency symptoms.

These policies, in combination with negative perceptions about the quality of care available to them, dissuaded participants from seeking medical services. Deon (Black) described new and unfamiliar “breathing problems” and rising “blood pressure” in IMU, but felt that seeking medical attention would be useless:

It’s pointless for me to knock on the window and ask the nurse, “Hey, nurse, do this.” Because every time I knock on the window–it is pointless because the only thing the DOC wants is money. It is money… I think people in the cell should be important… And it’s a long time but I’d just rather wait till I get out.

Later in the interview, Deon links his rising blood pressure to his isolation: “I never had blood pressure problems until I went to this IMU.” Because Deon does not expect to be treated with care or dignity, he avoids medical treatment. As a result, his new breathing issues and rising blood pressure went unnoticed by medical staff, and Deon did not find out the cause.

Blake (white), described experiencing unfamiliar physical health symptoms in the IMU, for which he was also hopeless about receiving any medical assistance:

I’ve been told I have a heart murmur, but for, like, last two weeks…I’ve been feeling my heart, like, feeling weird like it flutters once in a while…[I] just don’t tell nobody…because they won’t do nothing about it unless you’re actually having a heart attack, or unless you declare a medical emergency…they’ll pull you out, take your vitals, and then charge you 4 bucks… If I have a heart attack or don’t have a heart attack, it don’t matter.

Not only did Blake, like Deon, doubt whether a prison medical provider would believe him and try to help him, but he was further dissuaded from seeking treatment by the $4 institutionally-imposed cost for non-emergency treatment. Four dollars is arguably worth much more in prison that it would be even to a destitute person on the outside, and worth more still to someone in the IMU. Under WADOC policy, people in IMU are only allowed to spend $10 per week on store items, such as coffee, pastries, and deodorant. The $4 medical fee would absorb nearly half of this weekly spending cap. Blake might have had clinically insignificant, subjective palpitations, or the onset of atrial fibrillation following an undiagnosed myocardial infarction; his confinement status rendered clarification functionally unavailable.

Like many other participants, Deon and Blake expressed a sense of futility about seeking medical assistance while in the IMU, dissuaded by bureaucratic hurdles from perceived dismissiveness and indignity (exemplified in the problem of dual loyalty [67]) to actual costs of care. Futility, in turn, led to non-evaluation of emerging medical problems. Still, Deon and Blake expressed a passive acceptance of their situation: “it’s pointless,” and “it don’t matter.” This hopelessness reflects a precarity unique to solitary confinement: wondering whether medications would be provided and refills renewed, whether the severity of ailments would be acknowledged, and whether medical emergencies would be addressed or, instead, treated as security threats. As our participants’ experiences suggest, solitary confinement carries the additional punishment of substandard access to health care.

#### Exacerbating musculoskeletal pain

Participants spoke frequently about one specific, chronic ailment in solitary confinement: musculoskeletal pain. The experiences of people in solitary confinement with chronic musculoskeletal pain reveal how the prior two categories of symptoms we analyze, those associated with deprivation conditions and those associated with deprivation policies in solitary confinement, interact to exacerbate physical health problems. While participants attributed their musculoskeletal pain to a range of causes from physical injury to arthritis, bursitis, and sciatica, they consistently experienced this pain as untreated and interfering (physically and mentally) with even those few, limited activities available to them in solitary confinement.

For instance, Victor (Latino) described his frustrations with attempts to get care, let alone relief, from the pain of his sciatica:

I’ve been told I have nothing wrong with me, but I have been hurt, and they took x-rays of my back, and they found that the disks are in there or something that’s triggering some nerves. And I still got a little bit of time left, and they just opened up an Ibuprofen right now. And that stuff doesn’t work. So, what can you do?

Victor’s medical file highlights persistence of chronic pain in his back and hips and notes that he avoided sitting down for longer than 5–10 minutes. Not only did participants describe untreated pain, but they described the anxiety associated with the lack of treatment. Isaac (Black/Latino) described how he experienced both quad and hamstring pain in the IMU, and how this escalated his physical health concerns: “I’ll start thinking like oh, I’m laying in bed too much. Maybe my muscles are starting to rot, you know, eating on themselves.” In a similar sentiment, Tim (white) stated, “My body is like–I can’t explain it. Like my skeleton, feels like my skeleton’s broken or something.” While Victor must bear persistent pain and the anxiety that he will likely have to continue to suffer, Isaac and Tim’s experiences are more reflective of somatization, or the expression of psychological distress through physical symptoms [69]. These participants highlight the complex comorbidity between musculoskeletal pain and mental health in isolation, an inverse experience of physical pain. Tyler (white), discussing his scoliosis, made a direct connection between his untreated pain and his mental health: “Mental health and things that go through your head just because of this, when you got pain shooting up into your brain, and you guys aren’t fixing it.”

Pain and anxiety, in turn, interfered with other aspects of IMU existence. Craig (white) described how an untreated knee injury was causing him “moderate to severe pain,” in combination with anxiety about how he would re-enter society when released directly from solitary confinement; together these experiences interfered with his everyday activities, including his ability to communicate with his family. “I was in the middle of actually writing my mom a letter, and I was going to tell her about, you know, they still haven’t done anything with my knee…I couldn’t write the letter anymore. I just got so mad. I was so mad I really couldn’t even focus on anything.” Craig’s medical file affirms his complaint, documenting knee swelling and chronic extension tendonitis, but also indicating no abnormalities were found.

People living in solitary confinement are left with very few options to effectively manage persistent pain, which appears to foster more maladaptive behavior, such as rumination, stress, and despair, within a highly restrictive and stimuli-depleted environment [68–71]. Along with bearing the institutional monotony, medical precariousness, and procedural strictures of solitary confinement, one’s own body becomes a challenge to withstand [72, 73].

### Racial/Ethnic disproportionalities

We now turn to reporting the race and ethnic disparities in the Washington state prison population overall (compared to the statewide adult population), and in solitary confinement specifically (compared to the general prison population). These disparities suggest that the various mechanisms by which solitary confinement impacts health and well-being are likely to be disproportionately experienced across race and ethnic lines.

**Fig 1:**
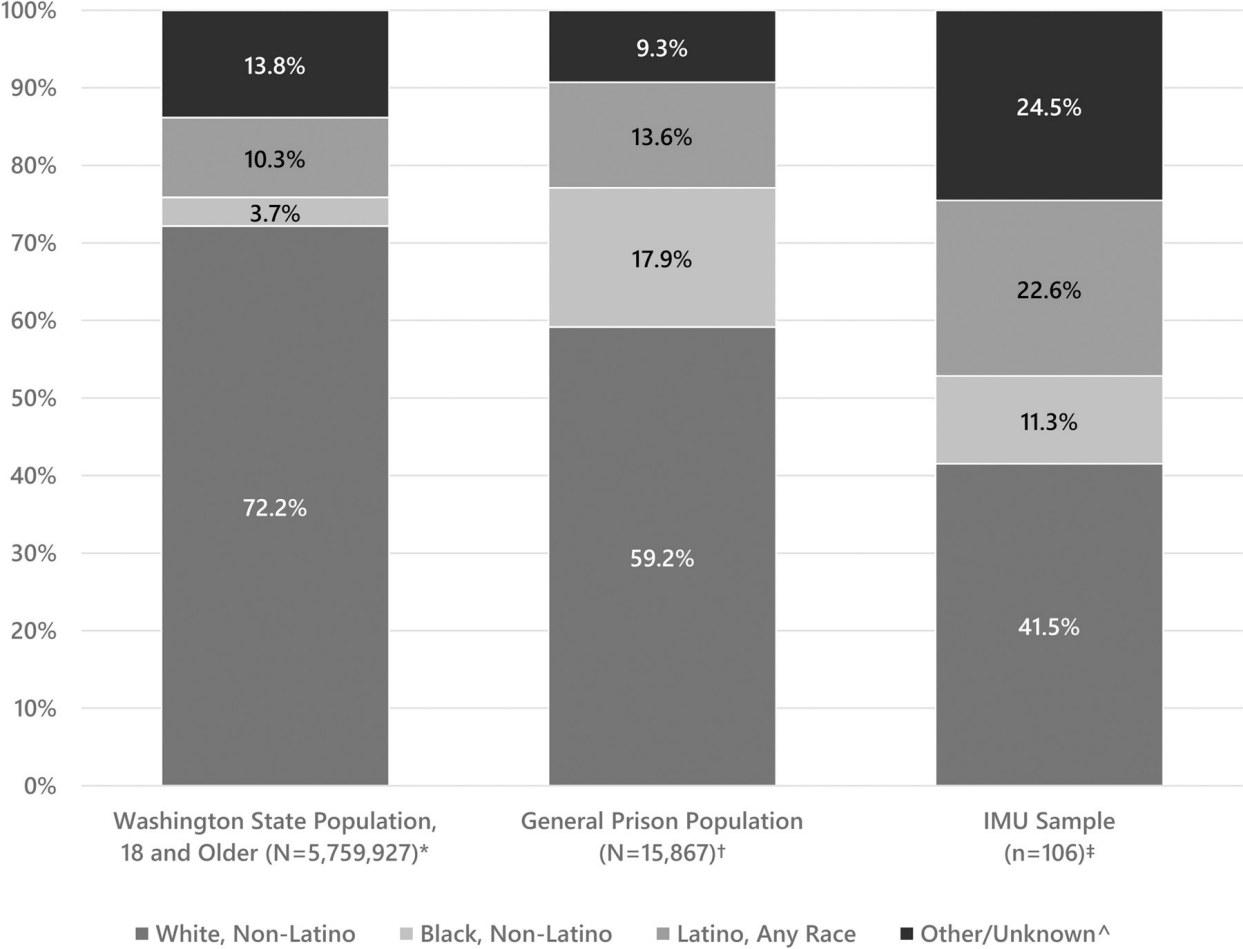
Racial and ethnic composition of IMU sample, general prison population, and Washington State, 2017. ^*^ U.S. Census Bureau, Population Division. Annual Estimates of the Resident Population by Sex, Age, Race, and Hispanic Origin for the United States and States: April 1, 2010 to July 1, 2017. 2018 Jun. ^**†**^ Authors’ calculations. The total prison population file included 17,943 individuals in DOC prison custody on July 1, 2017. For comparison purposes, the “general prison population” excludes those returned to prison on violations of release or sentence conditions, those in an IMU unit on the index date, and those on a maximum custody status (n = 1,970), as well as those in the IMU sample (n = 106). ^**‡**^ No significant differences in racial/ethnic composition were found between the IMU sample and larger IMU population on the index date using race/ethnicity data from DOC. These data reflect self-reported race/ethnicity during participant interviews. **^** Other/Unknown includes individuals of two or more races, Asian/Pacific Islander, Native American/Alaska Native, and unknown race/ethnicity information.

We analyze administrative data provided by WADOC and Census Bureau population estimates. Black, non-Latino individuals represented only 3.7% of adults in Washington state in 2017, but they comprised 17.9% of the general prison population [74]. Similarly, Latino individuals represented 10.3% of the statewide adult population, but 13.6% of the prison population. Conversely, both White, non-Latinos and Asian/Pacific Islanders, Native Americans, and mixed-race individuals (grouped within “Other/Unknown”) were somewhat under-represented in the general prison population relative to the statewide adult population (see Fig 1). Differences in racial and ethnic composition of the general prison population and the statewide adult population are statistically significant (*p* < .001; chi-square test for homogeneity).

Within prison walls, we find evidence of further racial and ethnic disproportionalities in housing placement. Comparing those housed in restrictive IMU confinement to those housed in the general population, we find that prisoners who self-identify as “Latino, Any Race” and “Other/Unknown” ethnicity are over-represented in IMU. To characterize the scale of differences in the racial/ethnic composition of the IMU and general prison populations, we calculated disproportionality, or prevalence, ratios as the proportion of each racial/ethnic group in a given population, divided by the proportion of that racial/ethnic group in the reference population. Here, Latinos are over-represented within the IMU participant group by a factor of 1.7 relative to their representation in the general prison population, and those grouped in the “Other/Unknown” category are over-represented in the IMU sample by a factor of 2.6, relative to the general prison population. Conversely, White, non-Latino individuals are under-represented in the IMU sample relative to the general prison population. Likewise, and in contrast to the gross disproportionality documented in the general prison population, Black, non-Latino individuals are moderately under-represented in the IMU sample, relative to the general prison population: 11.3% of the IMU sample identified as Black, non-Latino, compared with 17.9% of the general prison population. The difference in the racial and ethnic composition of those in long-term solitary confinement compared with the general population was statistically significant (*p* < .001; chi-square test for homogeneity).

## Discussion

A popular analogy likens prison to a chronic illness: it disrupts daily life, interrupts routines [72], spreads risk like a contagious disease [75], and models like an epidemiological problem [76, 30]. While the study of the physical effects of incarceration has developed over the last decade, there is a serious gap in the literature in understanding the experiences and outcomes of physical health in isolation. We are just beginning to understand the medical correlates of solitary confinement, their comorbidity with mental health, and overall implications for prisoners’ suffering [72]. Integrating surveys, interviews, BPRS scores, medical and disciplinary file reviews, and administrative data, the scale and array of our research represents one of the more robust studies of solitary confinement to date [20]. The multi-method research presented here offers a first step not only towards understanding some typical medical problems of solitary confinement, but also towards understanding the analytical challenges of an environment in which physical and psychological problems are immediately concomitant, and objective clarification is often unavailable.

We find that solitary confinement constitutes not just a mental but also a physical health risk. It exacerbates well-documented physical health “symptoms” of incarceration, from disruptions of daily life and routines, to undiagnosed, untreated, or mis-treated ailments [1, 30, 38]. These initial symptoms, in turn, produce other risks: to the extent respondents are accurately reporting weight fluctuations in solitary confinement, this physical symptom has detrimental health implications; weight fluctuation, itself, is associated with adverse cardiovascular and psychological outcomes [77, 78]. Likewise, musculoskeletal pain increases multimorbidity, and its sequelae are tightly unified in their impact on disability [79].

These health concerns likely have a grossly disparate impact on communities of color: just as incarceration is a health stratifying institution for prisoners, their families, and communities, so, too, does solitary confinement appear to exacerbate racial health inequities. While we find that Black, non-Latino individuals are moderately under-represented in the IMU sample, relative to the general prison population, we find that Latino and Other/Mixed Race prisoners are disproportionately over-represented in solitary confinement in WADOC, just as other studies have documented disproportionately high representations of racial and ethnic minorities in other states’ uses of solitary confinement [22, 41, 43]. We further find that prisoners of all races describe similar physical health challenges and complaints while in solitary confinement. In sum, people of color face a disproportionate risk of being placed in solitary confinement; such racial disparities, in turn, mean that the physical health symptoms associated with, or possibly caused by, these conditions of confinement are likely to fall disproportionately on certain groups. Though we do not explore other risk factors for over-representation in solitary confinement in this paper, we and others have documented serious mental illness [20, 80], transgender identification [81], and pregnant women [82] as particularly vulnerable to both incarceration and solitary confinement, suggesting additional sub-groups who might face disproportionate and unique risks of physical health problems in solitary confinement.

If anything, the evidence we present here understates the prevalence and intensity of the symptoms we document. First, Washington State is a progressive system actively engaged in both limiting the application and the duration of solitary confinement and developing measures to mitigate its harmful effects, from better mental health training for correctional staff to more sustained group contact for prisoners in IMUs; conditions, and their physical effects, are undoubtedly worse in many, if not most, other states [20, 42, 44]. Second, the BPRS somatic concerns scores we present focus on the two weeks prior to assessment, so likely underrepresent the cumulative incidence of somatic concerns in the study sample over time. Third, our exceptionally large random sample size for an in-depth, mixed methods study of a solitary confinement population was still not powered to establish statistically significant differences between interview subjects in the IMU in year one (2017) and those out of the IMU in year two (2018)–otherwise important comparison groups for understanding differences in either somatic concerns measures, or physical symptom specifications. Fourth, both the Washington state population and state prison population have proportionately more white people than some other states and prisons, where racial disparities in both prison and solitary confinement may be even more significant.

While our findings do not establish either how prevalent the symptoms and mechanisms of suffering we specified are among people in solitary confinement, as compared to the general prison population, or whether solitary confinement in fact directly causes these symptoms, recent research suggests that at least some of the symptoms our respondents reported, like hypertension, are significantly associated with long-term isolation [83, 45]. Although the evidence is clear that solitary confinement poses serious health risks [54, 45], our research highlights the importance of continuing to document and analyze these risks, especially from a multi-method perspective triangulating administrative population-level data with objective scales like the BPRS, subjective descriptions of experiences from surveys and interviews, and corroboration from medical file reviews.

First, documenting physical health problems provides a critical means to elucidate the severity of deprivations in treatment, environmental conditions, and exercise and nutrition [84, 85] inherent in solitary confinement. If incarceration is experienced fundamentally through control and restriction of the body, this is all the more true in solitary confinement, where prisoners are subjected to extreme forms of control while being entirely reliant on others for accessing basic necessities, from food to healthcare. Our participants experienced the deprivations of solitary confinement as exacerbating their health problems, which shaped their health experiences as punitive. Otherwise medically trivial conditions quickly become grave in solitary; “dandruff” can become a bleeding scalp wound, a four-dollar co-payment blurs the difference between subjective palpitations and an unstable arrhythmia, and unused muscles “rot.” Physical suffering reveals itself to be a crucial dimension of experience in solitary confinement.

Second, to the extent physical symptoms, in particular, are more familiar, more readily labeled, and less stigmatized than mental health issues, they may provide a window into other, less physically tangible pains of confinement, in solitary or elsewhere [84, 85]. The visuality of spectacular forms of suffering in carceral institutions is only made possible by and through mundane phenomenon that our participants elucidate through their discussions of everyday physical experiences [86]. Indeed, attending to people’s physical health in solitary confinement reveals the irreducible relationship between the body, mental health, and highly restrictive conditions of confinement. Whether they exercise to the point of physical debilitation to keep their minds busy, refuse to eat because they do not trust their food is safe, or avoid medical care out of a hopelessness of being treated with dignity, the physical and psychological are intimately bounded in people’s experiences in prison. Examining physical suffering in solitary confinement, then, becomes a tool for understanding suffering in prison more broadly, and especially the comorbidity of physical and mental suffering.

Third, the challenges we document in identifying and specifying physical symptoms in solitary confinement reveal not just the interrelationship between symptoms, conditions, and policies, but institutional mechanisms exacerbating both the identification and treatment of physical problems in prison. In many cases, our respondents had no hope of establishing what was physically wrong with them, let alone whether the conditions of their confinement caused the physical ailments, because they either could not get or avoided medical treatment. While both community standard and continuity of care is an issue in prison generally [67], solitary confinement widens these service gaps. The phenomenon of dual loyalty, which describes how the patient-provider relationship within prison can be subsumed by correctional directives of control and mistrust of incarcerated people [67], is acutely relevant in the context of solitary confinement, where both control and mistrust are especially prevalent [87, 88].

In sum, examining solitary confinement and documenting its affects provides an important magnifying lens for understanding prison and its affects more broadly, not only in elucidating the mechanisms of harm, but also in developing responses to mitigate these harms. Ninety-five percent or more of all prisoners will eventually return home to our communities [4, 5]; and many will have spent time in solitary confinement. Nearly one-in-five people in prison spends time in solitary confinement each year, and one-in-ten spends 30 days or more in these conditions [3]. These numbers will only increase in the face of the global COVID-19 pandemic, which has justified facility-wide “lockdowns,” imposing restrictions similar to those in solitary-confinement, in prisons across the United States, as well as actual solitary confinement placements for infected and exposed prisoners [89]. To the extent that solitary confinement undercuts treatment and care in and beyond prison, it undermines the public health of those incarcerated and those returning to our communities.

## Supporting information

S1 Text IMU survey. (PDF)

S2 Text Interview instrument. (DOC)

S1 Checklist Consolidated criteria for reporting qualitative studies (COREQ): 32-item checklist. (DOCX)

S1 Quotations (DOCX)
