# Supplementary material for: The body in isolation: The physical health impacts of incarceration in solitary confinement
Source: PLoS One. 2020 Oct 9;15(10):e0238510. doi: 10.1371/journal.pone.0238510 (PMC7546459; doi:10.1371/journal.pone.0238510)
Supplement: S1 Text — (PDF) [file pone.0238510.s001.pdf]

IMU Prisoner Survey

This survey is part of a study of conditions of confinement in Washington State Intensive Management Units. We are interested in understanding your experiences in the IMU. Based on the information you provide, we hope to be able to make recommendations about improving conditions of confinement in the IMU and about reducing the use of segregation throughout Washington State, as well as in other states. ***Please do not write your name on this survey****, and we will not reveal any identifying information about you in our research or to the Washington Department of Corrections.*

I. About Your Time in IMU (Intensive Management Unit)

First we would like to learn about where you are housed and how long you have been in this unit. For each question below, please write your response in the space provided or fill in the circle that matches to your answer.

1. Where are you currently housed? (Specify the prison unit, please)

   ____

2. How long have you been in prison?

   - O Less than one year
   - O Between 1 to 2 years
   - O Between 2 to 4 years
   - O Between 4 to 7 years
   - O Between 7 to 10 years
   - O 10 or more years

3. How long have you been housed here in this IMU (Intensive Management Unit)?

   ____

4. Why you were placed in the IMU?
   - O Extreme protection needs
   - O Violent or disruptive behavior
   - O Residential treatment for mental illness
   - O Pending transfer or in transit
   - O Considered an escape risk
   - O Under investigation for infraction
   - O Poses threat to self, staff, other offenders, or property

5. In your own words, can you tell us more about why you were place in this housing unit?

____

____

____

____

   a. Did you have an intake assessment before being placed in the IMU?

      ○ Yes ○ No

   b. If yes: What was it like? Who did you talk to?

____

____

____

6. Have you had any status reviews about your placement here? ○ Yes ○ No

   a. If yes: When? About how long ago?
      ____

   b. Have you had more than one review? How many?
      ____

7. Is this your first placement in this IMU? ○ Yes ○ No

   a. If no: How many times have you been in this IMU? ________________________

8. Have you been housed in other IMUs? ○ Yes ○ No

   a. If yes: Where? ____

   b. When? (Month/Year) ____

   c. For how long? ____

   d. Why? ____

      ____

9. While you have been housed in the IMU, have you seen changes in any of the following conditions:

	YES	NO
a) Health care for prisoners	O	O
b) Housing conditions	O	O
c) Food quality	O	O
d) Correctional officers' attitudes towards prisoners	O	O
e) Access to counselors or other mental health care	O	O
f) Length of time prisoners spend in IMU	O	O
g) Number of other prisoners in IMU	O	O
h) Amount of violence in IMU	O	O

10. Are there any other changes you have seen in the IMU housing unit you would like to tell us about?

____

____

____

11. Have you ever tried to challenge your placement here? O Yes O No

a. If yes: Why? ____

____

____

II. Basic Conditions of Confinement

Now we would like to learn more about your day-to-day activities and experiences in IMU.

12. On a daily basis in IMU, do you:

	YES	NO
a) Talk to other prisoners	O	O
b) See staff	O	O
c) See visitors	O	O
d) Leave your cell	O	O
e) See medical staff	O	O
f) Shower/bathe	O	O
g) Read or write	O	O

	YES	NO
h) Sleep	O	O
i) Get searched or pat down	O	O
j) Have cell searched	O	O
k) Wait for prisoner counts	O	O
l) Eat three meals	O	O
m) Pray or read religious text	O	O
n) Watch television	O	O
o) Exercise	O	O
p) Receive medication	O	O
q) Send mail	O	O
r) Receive mail	O	O

13. Can you describe a day in the IMU: what you do from the time you wake up until you go to sleep?

____

____

____

____

____

14. How often do you do each of the following:

	**Daily**	**Once a week**	**Once a month**	**Never**
a) Talk with other prisoners	O	O	O	O
b) See staff	O	O	O	O
c) Have visitors	O	O	O	O
d) Leave your cell	O	O	O	O
e) See medical staff	O	O	O	O
f) Shower/bathe	O	O	O	O
g) Read/write	O	O	O	O
h) Sleep	O	O	O	O
i) Get searched or pat down	O	O	O	O
j) Have cell searched	O	O	O	O
k) Wait for prisoner counts	O	O	O	O
l) Eat three meals	O	O	O	O
m) Pray or read religious text	O	O	O	O
n) Watch television	O	O	O	O
o) Exercise	O	O	O	O
p) Receive medication	O	O	O	O
q) Send mail	O	O	O	O
r) Receive mail	O	O	O	O

15. Compared to **general population housing**, how would you rate the conditions in IMU?
   - O <u>Much better</u> in IMU
   - O <u>Slightly better</u> in IMU
   - O No difference in IMU
   - O <u>Slightly worse</u> in IMU
   - O <u>Much worse</u> in IMU

16. Other people have described changes in themselves, after spending time in prison or in maximum security. In the time you have been here, have you experienced any changes in yourself?
    O Yes O No

    If yes, please describe them:

    ____

    ____

    ____

    ____

17. When do you expect to be released from IMU? ______________________________

18. What do you have to do in order to be released from IMU?

    ____

    ____

    ____

<u>III. Health and Well-being</u>

In this section of the survey, we would like to ask you about your health and well-being. This information will be used to describe prisoners in IMU *as a group*. <u>Your individual responses will not be shared with anyone.</u>

19. To begin, how would you rate your overall health?
   - O Poor
   - O Fair
   - O Good
   - O Very Good
   - O Excellent

20. Have you had any health problems in prison, where you needed professional care?
O Yes O No

21. Have you ever been told by a medical professional that you have one or more of the following conditions (check all that apply):

a) Diabetes	O YES	O NO
b) Congestive Heart Failure	O YES	O NO
c) Heart Disease (heart attack or angina)	O YES	O NO
d) History of a stroke	O YES	O NO
e) COPD (chronic bronchitis or emphysema)	O YES	O NO
f) Dementia of Mild Cognitive Impairment	O YES	O NO
g) Hearing Impairment	O YES	O NO
h) Arthritis	O YES	O NO

22. Have you fallen in the last 3 months, and gotten hurt? O Yes O No

    a. If yes, how many times? ____

23. Do you use a walker, cane or wheelchair? O Yes O No

24. Do you have trouble with any of the following daily activities:

a) Feeding yourself	O YES	O NO
b) Dressing yourself	O YES	O NO
c) Bathing yourself	O YES	O NO
d) Getting from your bed to a chair, or from a chair to standing	O YES	O NO
e) Using the toilet in your cell	O YES	O NO

25. How often do you see a doctor? ___________________________________

    a. Nurse? ____

    b. Therapist/Mental Health professional? ______________________________

    c. Dentist? ____

    d. Other medical professional? ____________________________________

26. Do you take any medications? O Yes O No

27. Have you ever tried to harm yourself in the IMU? O Yes O No

28. Have you ever felt unsafe in the IMU? O Yes O No

29. Do you feel more or less safe in the IMU than in the general prison population?
- ○ More safe in IMU
- ○ No difference in safety
- ○ Less safe in IMU

30. Do you have any other medical conditions you would like to tell us about?

____

____

____

IV. Background Information

Finally, we would like to ask you a few questions about yourself that will help us to interpret the results. We will use this information only to group you with others who are like you to see whether your views are similar.

31. What is your gender?
- ○ Male
- ○ Female
- ○ Other: ____________________

32. What is your marital status?
- ○ Married
- ○ Single (Never married)
- ○ Separated
- ○ Has a significant other
- ○ Divorced
- ○ Widowed

33. How old are you? ____________________

34. Do you have any children? ○ Yes ○ No

   a. If yes, how many? ____________________

35. What is the highest level of school you have completed or the highest degree you have received?
- ○ 8th grade or less
- ○ 9th to 11th grade
- ○ 12th grade (High school diploma/GED)
- ○ Some college, but no degree
- ○ Associate's degree
- ○ Bachelor's degree
- ○ Master's degree
- ○ Professional or doctorate degree

36. What race or ethnicity do you consider yourself? (Please choose all that apply)

- O Black/African-American
- O White/Caucasian
- O Hispanic or Latino/a
- O Asian/Pacific-Islander
- O Native American
- O Middle Eastern
- O Other: ___________________

Please feel free to add additional details about things we asked about, or to make suggestions about other things we should have asked about. You can also continue on to the back of the page if needed.

____

____

____

____

____

____

____

____

____

____

____

____

____
