# Supplementary material for: The body in isolation: The physical health impacts of incarceration in solitary confinement
Source: PLoS One. 2020 Oct 9;15(10):e0238510. doi: 10.1371/journal.pone.0238510 (PMC7546459; doi:10.1371/journal.pone.0238510)
Supplement: S2 Text — (DOC) [file pone.0238510.s002.doc]

**Study of Segregation Units in**

**Washington State Prisons:
Interview Instrument for Prisoners**

**Department of Criminology, Law and Society**

**
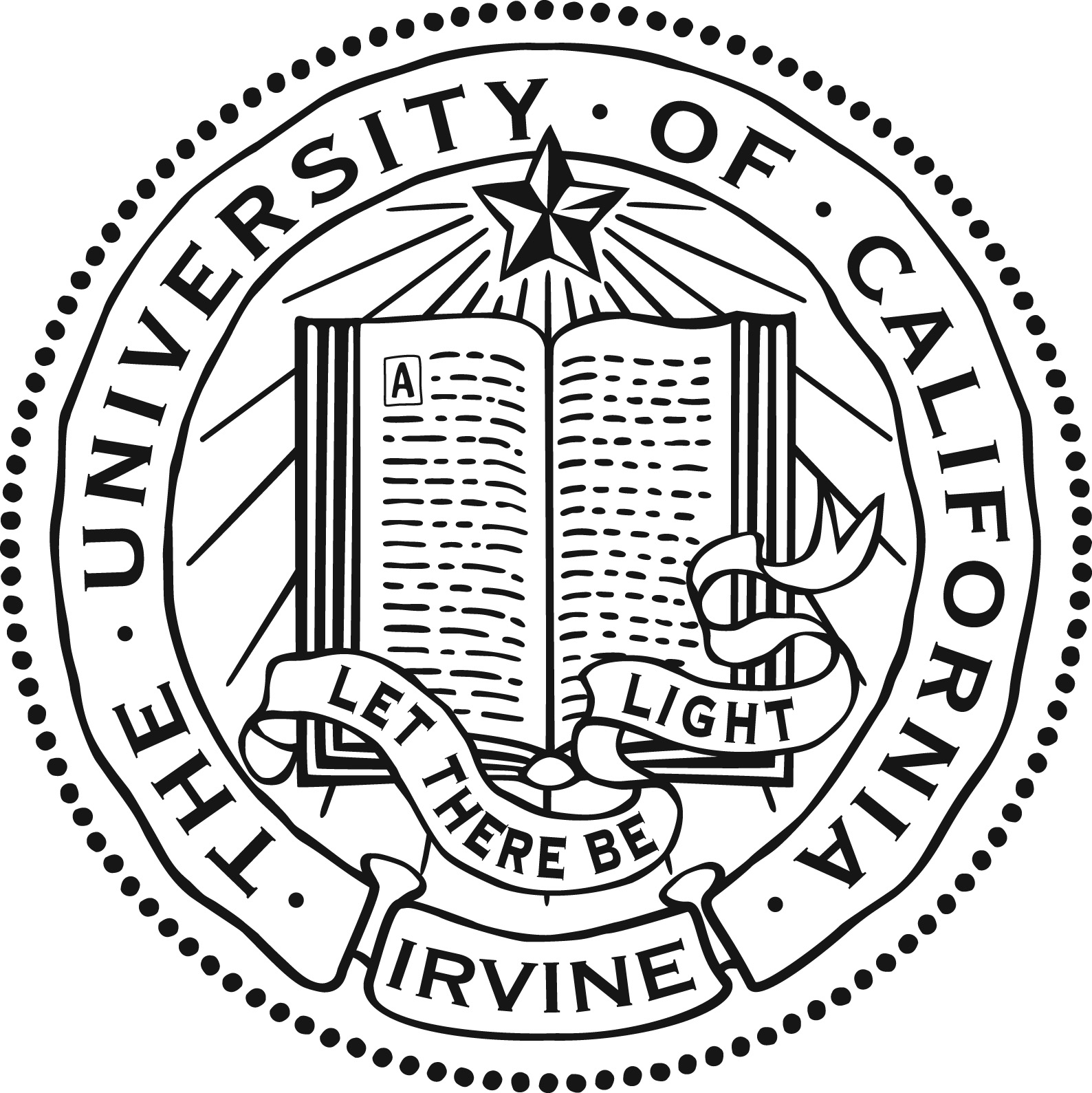
**

##### Interview Start Time: ________

| **A. INTRODUCTORY QUESTIONS** | | | | | | | | |
| --- | --- | --- | --- | --- | --- | --- | --- | --- |
| *Please remember that I will never ask you about any future intentions to harm yourself or others during this interview. I will also never ask you about any criminal activity, or activity that is prohibited by the WADOC. If you volunteer that information, I may be forced to report it to the appropriate authorities. I’d like to begin by asking you a little bit about yourself and your current living situation here at this prison.* | | | | | | | | |
| 1.  What is a day like for you here?  *Probe: What do you do when you wake up? Then what do you do?*  1a. Could you describe your cell to me? | Notes: | | | | | | | |
| 2. [BPRS 13]  In the IMU, how often do you get to shower and change your clothes? Are you able to take “bird baths” in your cell on non-shower days? Do you eat regular meals?  ***Important to Note:*** *Hygiene/Appearance visual standard as well as context* | 1 | 2 | | 3 | 4 | 5 | 6 | 7 |
| Notes: | | | | | | | | |
| 3.  How long have you been in this IMU? | Notes: | | | | | | | |
| 4.  Why were you placed in this IMU?  *Probe: If they say “don’t know,” ask follow up about reason told to them* | Notes: | | | | | | | |
| 5.  Do you remember if you had an intake assessment when you moved to this IMU? If yes, what was it like? | YES | NO | Notes: | | | | | |
| 6.  Have you had any classification reviews about your placement in the IMU?  *Probe: If yes, when was the last one? What was it like?* | YES | NO | Notes: | | | | | |

| **B. QUESTIONS ON CONDITIONS OF CONFINEMENT** | | | |
| --- | --- | --- | --- |
| *Now I’d like to ask you some questions about some of your experiences in solitary. I understand this might be difficult, so please tell me if you would rather not answer.* | | | |
| 7.  Do you participate in any programming here in this unit? If yes, what?  *Probe: What topics does your OCP cover?* | YES | NO | Notes: |
| 7a. *[If no to above]*  If no programming, why not?  *Probe:* *If they can’t identify any programming, ask about kinds of programs: classes outside of cell, A2A, in-cell coursework (OCP), etc* | Notes: | | |
| 8.  Of the programming you just described, is there any that has been useful to you?  *Probe: If yes, what has been the most useful and why?* | YES | NO | Notes: |
| 9.  Is there any programming that hasn’t been useful? Why? | YES | NO | Notes: |
| 10.  Have you ever refused to participate in any programming in the IMU?  *Probe: If yes, what programming? Why?* | YES | NO | Notes: |
| 11.  (*If yes to 10*) What happened after you refused programming?  (*If no to 10*) What would happen if you did refuse programming? | Notes: | | |
| 12.  What programming do you wish the IMU had?  *Probe: Why? How would it help you?* | Notes: | | |
| 13.  Have you been housed in other IMU’s?  *Probe: If yes, where and when? Why? How did it compare to this one?* | YES | NO | Notes: |
| 14.  Have you ever tried to challenge your placement in this IMU? Why or why not?  *Probe: If yes, what’d you do? [Get story}* | YES | NO | Notes: |
| 14a. *[If no to above]*  If not, do you know how you (or any IMU prisoner) would challenge their IMU placement? What would they do? | Notes: | | |
| 15.  When do you expect to be released from the IMU? | Notes: | | |
| 16.  What do you have to complete in order to be released? | Notes: | | |
| 17.  What do you think the transition out the IMU will be like? | Notes: | | |
| 17a.  Have you heard about any specialized units, to help transition out of the IMU? *(If yes, follow up)* What is that unit like? | YES | NO |  |

| **C. QUESTIONS ON PROBLEMS IN IMU** | | | |
| --- | --- | --- | --- |
| *Next, I’d like to ask you about any problems you might have had while housed in this IMU…* | | | |
| 18.  Have you ever filed a grievance while housed in the IMU?  *Probe: If yes, what?* | YES | NO | Notes: |
| 18a.  *(If yes to above)* Did you receive a response to your grievance? What did it say? How long did it take? | YES | NO | Notes: |
| 19.  Have you ever sent a kite to administration while in the IMU?  *Probe: If yes, for what?* | YES | NO | Notes: |
| 19a.  *(If yes to above)* Did you receive a response to your kite? What did it say? How long did it take? | YES | NO | Notes: |
| 20.  What is the hardest thing about being in the IMU? [*THINK: BPRS opening*] | Notes: | | |
| 21.  What is the easiest? | Notes: | | |
| 22.  How often do you talk with other prisoners? | Notes: | | |
| 23.  Can you describe your relationship with other prisoners?  *Probe: Are you close with anyone?* | Notes: | | |
| 24.  Can you tell me about the (prison) politics here in this IMU? What about the rest of the prison? | Notes: | | |
| 25.  Have you had any problems with other prisoners? *If yes, what kinds?* | YES | NO | Notes: |
| 26.  Do you receive visits here?  *If yes, how often and from who?* | YES | NO | Notes: |
| 27.  Have you had any trouble with the visitor policy in the IMU?  *If yes, what happened?*  *Probe: Non-relative visitors, banned visitors?* | YES | NO | Notes: |
| 28.  Have you experienced any other problems here in this IMU? If so, what are they? What’s bothersome?  *Probe: What has been your biggest problem? And why?*  [*THINK: BPRS opening*] | YES | NO | Notes: |

| **D. QUESTIONS ON HEALTH** | | | | | | | | | | |
| --- | --- | --- | --- | --- | --- | --- | --- | --- | --- | --- |
| *Now that we have talked about the different challenges here in the IMU, I’d like to ask you some more questions about your health and medical care while here in in the IMU….* | | | | | | | | | | |
| 29.  Have you ever sent a medical kite?  *Probe: If yes, for what?* | | | YES | | | NO | Notes | | | |
| 29a.  *(If yes to above)* Did you receive a response to your kite? What did it say? How long did it take? | | | YES | | | NO | Notes: | | | |
| 30.  How has your health been while you’ve been here in the IMU? | Notes: | | | | | | | | | |
| 31.  Have you noticed any changes in your health since you’ve been in the IMU?  What kinds? | YES | NO | | Notes: | | | | | | |
| 32.  How often do you get to visit a doctor?  *Reminder: Not just pill line* | Notes: | | | | | | | | | |
| 33.  How about a visit from a nurse?  *Reminder: Not just pill line* | Notes: | | | | | | | | | |
| 34.  And how often do you get treatment from a dentist? | Notes: | | | | | | | | | |
| 35.  Lastly, how often do you talk to a mental health counselor?  *Reminder: Not just pill line or just seeing them walk to another cell* | Notes: | | | | | | | | | |
| 36.  Do you take any medications? If yes, for what?  *Reminder: Please remember you can decline to answer any question* | YES | NO | | Notes: | | | | | | |
| 37.  Does it cost you money/Are you charged to see a doctor or dentist? How much does it cost? | YES | NO | | Notes: | | | | | | |
| 37a. *[If yes to above]*  Has this ever discouraged you from requesting medical or dental care you needed?  *Probe: If yes, what did you need? What did you do instead?* | YES | NO | | Notes: | | | | | | |
| 38. [BPRS 1]  Have you been concerned about your physical health in the last two weeks?  ***Important to note****: Interferes with daily activity; talked to others about health, specific body parts changing* | 1 | 2 | | | 3 | | 4 | 5 | 6 | 7 |
| Notes: | | | | | | | | | | |

| *Now that we’ve talked a little about your physical health, I’d like to talk with you more about how you’re coping in the IMU. These questions help us to understand the effects of the IMU on people’s health and well-being.* | | | | | | | | | | | | | | |
| --- | --- | --- | --- | --- | --- | --- | --- | --- | --- | --- | --- | --- | --- | --- |
| 39.  How do you feel most of the time in the IMU? | Notes: | | | | | | | | | | | | | |
| 40.  How do you cope with those feelings? | Notes: | | | | | | | | | | | | | |
| 41. [BPRS 2]  Thinking about just the last two weeks, have you felt worried or nervous?  ***Important to Note:*** *Physical effects, frequency, interference with daily activity* | 1 | | 2 | | 3 | | 4 | | | 5 | 6 | | | 7 |
| Notes: | | | | | | | | | | | | | | |
| 42. [BPRS 3]  Thinking about just the last two weeks, how has your mood been? Have you felt depressed?  ***Important to Note:*** *Able to switch attention; Loss of interest in enjoyable things, duration, interference with daily activity* | | 1 | 2 | | 3 | | 4 | | | 5 | 6 | | | 7 |
| Notes: | | | | | | | | | | | | | | |
| 43.  In the past, have you ever thought about harming yourself while in the IMU?  *Disclaimer: This is not* ***INTENT*** *to harm, and does not need to be disclosed to any WADOC authority* | | Notes: | | | | | | | | | | | | |
| 44.  In the past, have you ever harmed yourself (or tried to) while in the IMU?  *Disclaimer: This is not* ***INTENT*** *to harm, and does not need to be disclosed to any WADOC authority* | | Notes: | | | | | | | | | | | | |
| 45.  *(If yes)* Have you ever been punished for harming/trying to harm yourself?  *(If no)* Do you think you would be punished if you did? | | Notes: | | | | | | | | | | | | |
| 46. [BPRS 4]  Thinking about just the last two weeks, have you felt like life wasn’t worth living, at any point?  ***Important to Note:*** *Thoughts about suicide, up to actual suicide attempt* | | 1 | 2 | | 3 | | 4 | | | 5 | 6 | | | 7 |
| Notes: | | | | | | | | | | | | | | |
| 47. [BPRS 5]  Thinking about just the last two weeks, have you been thinking about past problems or things you are ashamed of?  ***Important to Note:*** *Frequency, Disclosure, Able to switch attention* | | 1 | 2 | | 3 | | 4 | | | 5 | 6 | | | 7 |
| Notes: | | | | | | | | | | | | | | |
| 48. [BPRS 6]  Thinking about just the last two weeks, have you felt irritable or angry? How did you show it?  ***Important to Note:*** *Started fights or arguments with staff, prisoners, or others; Hit anyone; Started confrontation* | | 1 | 2 | | 3 | 4 | | | 5 | | | 6 | | 7 |
| Notes: | | | | | | | | | | | | | | |
| 49. [BPRS 7]  Thinking about just the last two weeks, have you felt really good at all? Was there any reason? How long did it last?  ***Important to Note:*** *Euphoria versus good mood* | 1 | | 2 | 3 | | | | 4 | 5 | | | | 6 | 7 |
| Notes: | | | | | | | | | | | | | | |

| *We’re almost finished with this section. Lastly, we have some questions asking about things that other people have talked about experiencing in IMU’s.* | | | | | | | |
| --- | --- | --- | --- | --- | --- | --- | --- |
| 50. [BPRS 8]  So, is there anything special about you? Do you have special abilities or powers?  ***Important to Note:*** *Told others, acted on beliefs, frequency* | 1 | 2 | 3 | 4 | 5 | 6 | 7 |
| Notes: | | | | | | | |
| 51. [BPRS 10]  Have you ever heard any sounds or people talking to you or about you when there has been nobody around?  Do you ever have visions or see things others don’t see? What about smell odors others don’t smell?  ***Important to Note:*** *Interferes with daily activity, frequency, explanation* | 1 | 2 | 3 | 4 | 5 | 6 | 7 |
| Notes: | | | | | | | |
| 52. [BPRS 11]  Can anyone read your mind? Are thoughts put into your head that are not your own? Have you been receiving any special messages?  ***Important to Note:*** *Frequency, explanation, told others, Degree of conviction* | 1 | 2 | 3 | 4 | 5 | 6 | 7 |
| Notes: | | | | | | | |
| 53. [BPRS 12]  Have you done anything that seemed unusual or disturbing to others?  ***Important to Note:*** *Degree of attention* | 1 | 2 | 3 | 4 | 5 | 6 | 7 |
| Notes: | | | | | | | |

| **E. QUESTIONS ON PERCEPTIONS OF STAFF** | | | | | | | | | | | |
| --- | --- | --- | --- | --- | --- | --- | --- | --- | --- | --- | --- |
| *Next, I’d like to ask you some questions about the staff who work here in the IMU…* | | | | | | | | | | | |
| 54.  In general, how do you feel about the correctional officers?  *Follow-up: Why?* | | Positive | | Negative | No Opinion | | | Notes: | | | |
| 55.  Do you trust the correctional officers in this unit? Why or why not? | | YES | | NO | Notes: | | | | | | |
| 56.  Do you believe the CO’s in this unit… | |  | | | | | | | | | |
|  | a. …deliver all your mail to you? | YES | | NO | Notes: | | | | | | |
|  | b. …try their best to make sure you get shower/yard? | YES | | NO | Notes: | | | | | | |
|  | c. ... make sure you get all your meds correctly? | YES | | NO | Notes: | | | | | | |
|  | d. …would turn in a grievance form you sent? | YES | | NO | Notes: | | | | | | |
|  | e. …would protect you from other prisoners? | YES | | NO | Notes: | | | | | | |
|  | f. …would help you in a medical emergency? | YES | | NO | Notes: | | | | | | |
| 57.  While living in this IMU, would you say the correctional officers are watching you..?  *PROBE: Are they observing what you’re doing…?* | | All of the Time | | Most of  the Time | Occasionally | | | Rarely | | Never | |
| *Now let’s talk a little more about the other staff that work in the IMU….* | | | | | | | | | | | |
| 58.  What about the mental health staff? How do you feel about them?  *Follow-up: Why?* | | Positive | | Negative | No Opinion | | Notes: | | | | |
| 58a.  Do you trust the mental health staff in this unit? Why or why not? | | YES | | NO | Notes: | | | | | | |
| 59.  And what do you think about the medical staff?  *Follow-up: Why?* | | Positive | | Negative | No Opinion | | Notes: | | | | |
| 59a.  Do you trust the doctors/nurses in this unit? Why or why not? | | YES | | NO | Notes: | | | | | | |
| 60.  What about programming staff?  *Follow-up: Why?* | | Positive | | Negative | No Opinion | | Notes: | | | | |
| 60a.  Do you trust the programming staff in this unit? Why or why not? | | YES | | NO | Notes: | | | | | | |
| 61.  What about the warden and other administrators who run the prisons?  *Follow-up: Why?* | | Positive | | Negative | No Opinion | | Notes: | | | | |
| 61a.  Do you trust the warden and other administrators at this prison? Why or why not? | | YES | | NO | Notes: | | | | | | |
| 62.  Has any prison staff member ever helped you while you were in the IMU?  *Probe: c/o, admin*, *programming staff, medical staff. If so, how?* | | YES | | NO | Notes: | | | | | | |
| 63.  Have you had problems with any prison staff while housed in this IMU? *Probe: If yes, what?* | | YES | | NO | Notes: | | | | | | |
| 64.  Have you ever been disrespected by any prison staff in this IMU?  *Probe: If yes, how?* | | YES | | NO | Notes: | | | | | | |
| 65.  Have you ever received an infraction for something you didn’t do? Have you ever been infracted unfairly? | | YES | | NO | Notes: | | | | | | |
| 66.  Have you ever been verbally harassed by any prison staff in this IMU?  *Probe: If yes, how? Threats, names, racial slurs?* | | YES | | NO | Notes: | | | | | | |
| 67.  Have staff ever used force against you?  *If yes, probe for story* | | YES | | NO | Notes: | | | | | | |
| 68. [BPRS 9]  Thinking about just the last two weeks, have you felt like anyone was going out of their way to give you a hard time, or trying to hurt you?  ***Important to Note:*** *Frequency, degree of preoccupation* | | 1 | 2 | 3 | 4 | 5 | | | 6 | | 7 |
| Notes: | | | | | | | | | | | |

| **F. QUESTIONS ON PERSONAL SAFETY IN IMU** | | | | | | | |
| --- | --- | --- | --- | --- | --- | --- | --- |
| *Now I want to ask you about safety in this prison…* | | | | | | | |
| 69.  In general, how safe do you feel in this IMU? | | Very Safe | Safe | Unsafe | | Very Unsafe | |
| 70.  *Follow-up*: What things make you feel safe/unsafe? | | Notes: | | | | | |
| 71.  Do you feel safe from… | |  | | | | | |
|  | a. … verbal harassment from other prisoners? | YES | NO | Notes: | | | |
|  | b. … physical threats/assaults from other prisoners? | YES | NO | Notes: | | | |
|  | c. … verbal harassment from CO’s? | YES | NO | Notes: | | | |
|  | d. … physical threats/assaults from CO’s? | YES | NO | Notes: | | | |
|  | e. … yourself? | YES | NO | Note: | | | |
| 72.  Can you tell me about the time when you felt your safety was at greatest risk in the IMU?  *Probe: What made them feel at risk?* | | Notes: | | | | | |
| 73.  Do you feel more safe in the IMU, or in the General Population? Why? | | IMU | GEN POP | Notes: | | | |
| 74.  How often do you worry about your physical safety? | | All of the Time | Most of the Time | Occasionally | Rarely | | Never |
| 75.  Related to your safety, what do you worry about most here in this IMU?  [*THINK: BPRS opening*] | | Notes: | | | | | |
| 76.  In your opinion, what could be done to improve the safety of prisoners in this IMU? | | Notes: | | | | | |

| **G. QUESTIONS ON SEGREGATION REFORMS** | | | |
| --- | --- | --- | --- |
| *We’re almost finished. In the last few years, there has been a lot of attention on prison segregation units across the*  *country. As a result, many prisons have made policy changes, or reforms, in their segregation units. I’d like to ask*  *you some questions about what you know about reforms that are supposed to be going on here in Washington…* | | | |
| 77.  Are you aware of any current IMU  reforms?  *Probe: If yes, what reforms do you know about?* | Notes: | | |
| 77a.  **[IF NO - REFER BACK TO PREVIOUSLY DISCUSSED IMU PROGRAM FOR**  **CONCRETE EXAMPLE]** | | | |
| 78.  What is the purpose of these reforms? | Notes: | | |
| 79.  Are these reforms making things better for you?  *Probe: Why or why not?* | YES | NO | Notes: |
| 80.  Have there been surprising impacts from  these reforms? *If so, what kinds of things?* | YES | NO | Notes: |

| **H. DEMOGRAPHIC INFORMATION** | | | | | | | | | | | | | |
| --- | --- | --- | --- | --- | --- | --- | --- | --- | --- | --- | --- | --- | --- |
| *Finally, I’d like to ask some general questions about yourself …* | | | | | | | | | | | | | |
| 81.  How old are you? | Notes: | | | | | | | | | | | | |
| 82.  What gender are you?  *Remind it will be a written record* | MALE | | FEMALE | | | | | Other: | | | | | |
| 83.  What race are you? **[Don’t need to read responses]** | Black | | | White | | | | Hispanic/  Latino | | Asian | | | Other: |
| 84.  What is the highest grade you completed in school? **[Don’t need to read responses]** | [____Grade] | | | | [High School Graduate] | | | | | | [GED] | | |
|  | [Some College] | | | | [College Graduate] | | | | | | [Any Post-Graduate] | | |
| 85.  Are you married? | YES | NO | | | | Notes: | | | | | | | |
| 86.  Do you have any children? **[If yes]** How many? | YES | NO | | | | How many? | | | | | | | |
| 87.  How long have you been in prison? | Notes: | | | | | | | | | | | | |
| 88.  Are you currently or have you ever been a member of a prison gang?  *Reminder - you can choose not to answer any question* | NEVER | | | | | | | PREVIOUSLY | | | CURRENT | | |
| 89.  What about politics – where do you fall politically? | Liberal/Democrat | | | | | | Independent | | Conservative/  Republican | | | None | |
| 90.  Are you religious? | Notes: | | | | | | | | | | | | |
| *In this last bit, I have some different kinds of questions for you. These are just questions to get a feel for the general knowledge of prisoners housed in segregation…* | | | | | | | | | | | | | |
| 91. [BPRS 14a]  What’s the date today? | Notes: | | | | | | | | | | | | |
| 92. [BPRS 14b]  Who is the current president? | Notes: | | | | | | | | | | | | |
| 93. [SANS 22]  Can you count backwards from 100 by 7?  *(If less than 6^th^ grade)* Can you count backwards from 100 by 3? | [100] [93] [86] [79] [72] [65] [58] [51] | | | | | | | | | | | | |
|  | [100] [97] [94] [91] [88] [85] [82] [79] [76] | | | | | | | | | | | | |
| 94.  Can you spell the word **BRING** backwards? | Notes: | | | | | | | | | | | | |

| **I. CONCLUSION** | |
| --- | --- |
| *Thank you so much for speaking with me today. I have two final questions for you to wrap-up our interview…* | |
| 95.  If there was one thing that you’d want people to understand about life here in the IMU, what would it be? | Notes: |
| 96.  Is there anything I should have asked about your experiences, that you’d like to add? | Notes: |

**Interview End Time: __________**

**VISIBLY TURN OFF RECORDER**

| **ADDITIONAL CONSENTS** | |
| --- | --- |
| *Thank you again for agreeing to be interviewed today. Remember, everything you shared with me today will be kept confidential and made anonymous, so it will NOT be connected to your identity at all. Before we end, there are a few other kinds of information that would be very helpful for us to collect from you, that I need your permission for,*  *separate from the interview. I’ll go through each of them now with you….* | |
|  | **VERBAL PERMISSION TO FOLLOW-UP IN ONE YEAR**  (Interviewee name will be kept on a separate list, by UCI, to find & contact in one year) |
|  | **SIGNED FORM GRANTING PERMISSION TO ACCESS DOC HEALTH RECORDS**  (Form will be stored with UCI) |
|  | **SIGNED FORM GRANTING PERMISSION TO ACCESS DOC SUBSTANCE ABUSE RECORDS**  (Form will be stored with UCI) |

NOTES:

|  |
| --- |
|  |
|  |
|  |
|  |
|  |
|  |
|  |
|  |
|  |
|  |
|  |
|  |
|  |
|  |
|  |
|  |
|  |
|  |
|  |
|  |

**Brief Psychiatric Rating Scale** (Version 4.0)

IMU Facility Period of Assessment __Two Weeks____________

NA 1 2 3 4 5 6 7

Not Assessed Not Present Very Mild Mild Moderate Moderately Severe Severe Extremely Severe

*Rate items 1-14 on the basis of patient’s self-report during interview. Note items 7, 12, and 13 are*

*also rated on observed behavior during the interview. Mark “NA” for symptoms not assessed.*

***PROVIDE EXAMPLES:***

1. Somatic Concern NA 1 2 3 4 5 6 7

2. Anxiety NA 1 2 3 4 5 6 7

3. Depression NA 1 2 3 4 5 6 7

4. Suicidality NA 1 2 3 4 5 6 7

5. Guilt NA 1 2 3 4 5 6 7

6. Hostility NA 1 2 3 4 5 6 7

7. Elevated Mood NA 1 2 3 4 5 6 7

8. Grandiosity NA 1 2 3 4 5 6 7

9. Suspiciousness NA 1 2 3 4 5 6 7

10. Hallucinations NA 1 2 3 4 5 6 7

11. Unusual Thought Content NA 1 2 3 4 5 6 7

12. Bizarre Behavior NA 1 2 3 4 5 6 7

13. Self-neglect NA 1 2 3 4 5 6 7

14. Disorientation NA 1 2 3 4 5 6 7

Rate items 15-24 on the basis of observed behavior or speech of the patient during the interview.

15. Conceptual Disorganization NA 1 2 3 4 5 6 7

16. Blunted Affect NA 1 2 3 4 5 6 7

**-** **Unchanging Facial Expression** 0 1 2 3 4 5

The patient's face appears wooden, changes less than expected

as emotional content of discourse changes.

**- Decreased Spontaneous Movements** 0 1 2 3 4 5

The patient shows few or no spontaneous movements, does

not shift position, move extremities, etc.

**­- Paucity of Expressive Gestures** 0 1 2 3 4 5

The patient does not use hand gestures, body position, etc., as

an aid to expressing his ideas.

**- Poor Eye Contact** 0 1 2 3 4 5

The patient avoids eye contact or "stares through" interviewer

even when speaking.

**- Affective Nonresponsivity** 0 1 2 3 4 5

The patient fails to smile or laugh when prompted.

**- Lack of Vocal Inflections** 0 1 2 3 4 5

The patient fails to show normal vocal emphasis patterns, is

often monotonic.

**- Global Rating of Affective Flattening (BPRS 16)** 0 1 2 3 4 5

This rating should focus on overall severity of symptoms,

especially unresponsiveness, eye contact, facial expression,

and vocal inflections.

17. Emotional Withdrawal NA 1 2 3 4 5 6 7

18. Motor Retardation NA 1 2 3 4 5 6 7

- **Poverty of Speech** 0 1 2 3 4 5

The patient's replies to questions are restricted in the

amount,tend to be brief, concrete, and unelaborated.

**- Blocking**  0 1 2 3 4 5

The patient indicates, either spontaneously or with prompting,

that his or her train of thought was interrupted.

**- Increased Latency of Response** 0 1 2 3 4 5

The patient takes a long time to reply to questions; prompting

indicates the patient is aware of the question.

**- Global Rating of Alogia** 0 1 2 3 4 5

The core feature of alogia is poverty of speech

19. Tension NA 1 2 3 4 5 6 7

20. Uncooperativeness NA 1 2 3 4 5 6 7

21. Excitement NA 1 2 3 4 5 6 7

22. Distractibility NA 1 2 3 4 5 6 7

23. Motor Hyperactivity NA 1 2 3 4 5 6 7

24. Mannerisms and Posturing NA 1 2 3 4 5 6 7

Sources of information (check all applicable): Explain here if validity of assessment is questionable:

Patient Symptoms possibly substance-induced

Parents/Relatives Under reported due to lack of rapport

Mental health professionals Patient uncooperative

Chart Difficult to assess due to formal thought disorder

Other (e.g., police report) Other

Confidence in assessment Record information:

1 = not at all - 5 = very confident
