# Supplementary material for: The body in isolation: The physical health impacts of incarceration in solitary confinement
Source: PLoS One. 2020 Oct 9;15(10):e0238510. doi: 10.1371/journal.pone.0238510 (PMC7546459; doi:10.1371/journal.pone.0238510)
Supplement: S1 Checklist — (DOCX) [file pone.0238510.s003.docx]

S1 Checklist. Consolidated criteria for reporting qualitative studies (COREQ): 32-item checklist

| **No** | **Item** | **Guide questions/description** |
| --- | --- | --- |
| **Domain 1: Research team and reflexivity** |  |  |
| Personal Characteristics |  |  |
| 1 | **Interviewer/facilitator** | **Which author/s conducted the interview or focus group?**  *All authors listed, except Rebecca Tublitz and Thomas R. Blair, conducted interviews for this study.* |
| 2. | **Credentials** | **What were the researcher's credentials? *E.g. PhD, MD***  *At the time of interviews, research team credentials consisted of PhDs, JDs, MSs* |
| 3. | **Occupation** | **What was their occupation at the time of the study?**  *At the time of interviews, research team occupations consisted of Professor, Assistant Professor, and graduate students* |
| 4. | **Gender** | **Was the researcher male or female?**  *At the time of interviews, there were 9 females and 4 males on the research team.* |
| 5. | **Experience and training** | **What experience or training did the researcher have?**  *All interviewers underwent an extensive training process, including more than 20 hours of meetings to learn about conditions in Washington Department of Corrections (WADOC) Intensive Management Units (IMUs) and develop the interview instrument. Interviewers completed an additional 20 hours of a standardized training protocol for administering the BPRS in clinical settings: 16 hours of in-person symptom assessment training sessions with a leading expert in BPRS research—Dr. Joseph Ventura—in year one, and four hours of refresher training prior to the year-two interviews. Dr. Ventura conducted an interrater reliability analysis confirming trained raters met the minimum standard of an ICC=.80 or greater for the BPRS. This extensive training sought to ensure that the 13 team members (9 women and 4 men; 9 white and 4 non-white), all faculty or doctoral students with expertise in prisons, identified and addressed any pre-existing assumptions about the population being studied and minimized any possible bias as a result of inconsistent interpretation or application of questions and assessments.* |
| Relationship with participants |  |  |
| 6. | **Relationship established** | **Was a relationship established prior to study commencement?**  *Yes, study was facilitated by relationships cultivated between research PI and WADOC. Moreover, the pilot survey also provided the opportunity for potential participants to learn about the study and ask members of the research team questions before the time of interviews.* |
| 7. | **Participant knowledge of the interviewer** | **What did the participants know about the researcher? e*.g. personal goals, reasons for doing the research***  *Participants understood that research was being done to understand what life is like in the Intensive Management Units (IMU) in the WADOC, with special attention to the provision of programming and recent changes in IMU policy.* |
| 8. | **Interviewer characteristics** | **What characteristics were reported about the interviewer/facilitator? e.g. *Bias, assumptions, reasons and interests in the research topic***  *We have reported that at the time of interviews, all team members possessed some interest in prison research and/or had previously engaged in research with people in confinement settings or with people who were previously incarcerated. As also stated above, research team training consisted of discussion and addressing various team biases about the population.* |
| **Domain 2: study design** |  |  |
| Theoretical framework |  |  |
| 9. | **Methodological orientation and Theory** | **What methodological orientation was stated to underpin the study? *e.g. grounded theory, discourse analysis, ethnography, phenomenology, content analysis***  *We state that this particular study is oriented by constructivist grounded theory* |
| Participant selection |  |  |
| 10. | **Sampling** | **How were participants selected? *e.g. purposive, convenience, consecutive, snowball***  *Surveys were distributed to everyone on maximum custody status in a WADOC IMU.*  *Interview participants were randomly sampled. Specifically, during the summer of 2017, 106 people held in solitary confinement were selected for interviews from a randomly ordered list in proportion to the population of each IMU, accounting for 29 percent of the total population of 363 in each of the five units across the WADOC.* |
| 11. | **Method of approach** | **How were participants approached? e*.g. face-to-face, telephone, mail, email***  *All participants were initially approached face-to-face at cell front, where the purpose of the study was explained to them. If they were interested in learning more, they would be brought from their cell to a private (auditorily, but not visually) visiting area and formally consented prior to the initiation of interviews.* |
| 12. | **Sample size** | **How many participants were in the study?**  *106 interview subjects, 225 survey respondents, and 17,943 individual administrative records reflecting all those incarcerated in WADOC in 2017* |
| 13. | **Non-participation** | **How many people refused to participate or dropped out? Reasons?**  *The refusal rate of survey respondents was 38 percent (138 out of 363). The refusal rate of interview participants was 39 percent (67 out of 173 approached). Reasons were not provided by participants.* |
| Setting |  |  |
| 14. | **Setting of data collection** | **Where was the data collected? e*.g. home, clinic, workplace***  *Data was collected within five WADOC prison facilities. Interviews were conducted in confidential areas, like offices and legal visitation booths.* |
| 15. | **Presence of non-participants** | **Was anyone else present besides the participants and researchers?**  *Prison staff were required to monitor confidential areas visually, but not aurally.* |
| 16. | **Description of sample** | **What are the important characteristics of the sample? *e.g. demographic data, date***  *All participants were on maximum custody status and incarcerated in an Intensive Management Unit (IMU, or solitary confinement) at the time of the interview.* |
| Data collection |  |  |
| 17. | **Interview guide** | **Were questions, prompts, guides provided by the authors? Was it pilot tested?**  *Prior to interviews, paper surveys were distributed in-person (and collected on the same day) to all people on maximum custody status in the five state IMUs in the spring of 2017 (363 prisoners). In total, 225 prisoners (62%), responded, a response rate comparable to similar studies of prisoners. The interview instrument consisted of 96 numbered semi-structured questions (each containing a combination of yes/no questions and probing, open-ended follow-up questions) seeking elaboration on responses from the survey questions and also drawing from existing studies on prisons and prisoner experiences [66-70], including conditions of daily life (prior to and during isolation), perceived state of physical and mental health, access to medical treatment, and experiences with required programming in the IMU, among other topics.* |
| 18. | **Repeat interviews** | **Were repeat interviews carried out? If yes, how many?**  *Eighty people from our initial random sample, who were still incarcerated one year later, including those no longer housed in the IMU, were re-interviewed in 2018.* |
| 19. | **Audio/visual recording** | **Did the research use audio or visual recording to collect the data?**  *All participants consented to audio recording of interviews.* |
| 20. | **Field notes** | **Were field notes made during and/or after the interview or focus group?**  *Interview instruments provided space for researchers to take notes. All interviewers’ notes were scanned digitally. The PI also took extensive fieldnotes (via dictation) at the end of each day in a prison facility; these notes were transcribed and used for contextual background and reference.* |
| 21. | **Duration** | **What was the duration of the interviews or focus group?**  *Interviews ranged from 45 minutes to 3 hours.* |
| 22. | **Data saturation** | **Was data saturation discussed?**  *The team sought to achieve data saturation through an iterative process (surveys, interviews, re-interviews) and through the large scale of the random interview sample (one-third of the eligible population). Data saturation was further discussed during the coding process.* |
| 23. | **Transcripts returned** | **Were transcripts returned to participants for comment and/or correction?**  *No, this was not a realistic option given the movement of our participants in and out of the WADOC. It would also be near impossible to provide participants with copies of transcripts while ensuring their confidentiality, since all incoming and outgoing mail in prison is censored, and additional in-person visits beyond the interviews were beyond the budget and chronological scale of the project.* |
| **Domain 3: analysis and findings** |  |  |
| Data analysis |  |  |
| 24. | **Number of data coders** | **How many data coders coded the data?**  *Six team members, who also conducted interviews, engaged in an iterative and recursive coding process to generate a codebook from the data.* |
| 25. | **Description of the coding tree** | **Did authors provide a description of the coding tree?**  *Yes, the coding process generated a list of 214 codes, grouped into 11 major categories (e.g., Health) with sub-themes (e.g., physical health)* |
| 26. | **Derivation of themes** | **Were themes identified in advance or derived from the data?**  *Themes were derived from the data.* |
| 27. | **Software** | **What software, if applicable, was used to manage the data?**  *Atlas.ti* |
| 28. | **Participant checking** | **Did participants provide feedback on the findings?**  *No, this was not a realistic option given the movement and release of our participants in and out of prison and posed serious challenges to ensuring confidentiality.* |
| Reporting |  |  |
| 29. | **Quotations presented** | **Were participant quotations presented to illustrate the themes / findings? Was each quotation identified? e*.g. participant number***  *Yes, block quotes from at least two participants were presented in each sub-section of the findings.* |
| 30. | **Data and findings consistent** | **Was there consistency between the data presented and the findings?**  *Yes, after identifying instances when participants discussed physical health we then identified the institutional processes that effected their health. Here, participants’ physical health experiences were shaped by the conditions, policies, and resultant behavior adaptations of solitary confinement.* |
| 31. | **Clarity of major themes** | **Were major themes clearly presented in the findings?**  *Yes, organized around clearly titled sub-sections.* |
| 32. | **Clarity of minor themes** | **Is there a description of diverse cases or discussion of minor themes?**  *Yes, while our analysis focused primarily on the physical health effects of IMU we have also included discussion of instances when psychological health was experienced and expressed somatically. Overall, the framing is focused on identifying the range of physical symptoms experienced, inherently incorporating diverse cases.* |
