# Supplementary material for: The body in isolation: The physical health impacts of incarceration in solitary confinement
Source: PLoS One. 2020 Oct 9;15(10):e0238510. doi: 10.1371/journal.pone.0238510 (PMC7546459; doi:10.1371/journal.pone.0238510)
Supplement: S1 Quotations — (DOCX) [file pone.0238510.s004.docx]

**Supplemental Quotations**

1. **Deprivation Conditions**

| **Name, race/ethnicity** | **Quotation** |
| --- | --- |
| Santiago, Latino | The food doesn’t help with my routine. That’s what’s happening. I need to properly eat to do my routine. Before I had a heavy routine, where I would do, say 500 pushups, now I only can do 200… What happens is, to survive in the IMU, to sleep well, think well, relax, do all my things, I need to be well physically. And to be well physically, I have to do my exercises. And that’s what keeps me going mentally and spiritually. But if I don’t do my exercises, I start to think things and start to lose my mind. |
| Greg, Native American | [I] lost, like, 50 pounds…I work out every day. I mean, I'm only getting about maybe 2,800, 2,900 calories a day. Of that, a lot of it is breads and sugars, and I won't eat potatoes, I won't eat noodles, I won't eat bread, don't eat, like, sweets. I'll eat maybe 1,500, 1,800 calories a day, on top of working out. So, I'm losing weight rapidly. |
| Joseph, white | So, and they don't give you - they don't give you the - like options of buying conditioner or anything for your hair. You know? You can buy vitamins, but if you have a problem with your scalp, because it itches, because the soap is - the - it's very caustic and acidic. You know? Like if you look at the ingredients in - in the shampoo? It's not healthy for you. And - but you're forced to do it, and forced to use it, because it's the only kind that they have, and you have to wash your hair. |
| Joaquin, Latino | My skin dries a lot. We're not able to buy like good hygiene here. And the hygiene actually that they sell us is pretty much what they would provide you in main line if you were indigent. So, we have to pay in here for something that we get for free. And it sucks because even if you buy the lotion… like the heat of your hands just melts it. I have really sensitive skin. Like these areas start drying out real bad. So, that's every time I come to IMU. Sometimes I went as far as using butter, and it felt weird, but I had to do it. |

1. **Deprivation Policies**

| **Name, race/ethnicity** | **Quotation** |
| --- | --- |
| Tony, Native American & white | [I have a cyst] and it needs to be surgically removed. I filed all the grievances in the world at [other prison]- no action. I tentatively approached it here - they just kind of looked the other way. So I filed a lawsuit, so I'm not going to address it anymore, because I already exhausted everything. Now it's in the court. Now I'll get a preliminary injunction or a TRO, or a declaratory decree to have it taken care of. No more Medical. I'm tired of that. I tried it here. You have put in a Medical kite to have to be seen by Wednesday. Well, what - five for me isn't good enough? Five Medical kites explaining the situation. I mean, come on people! |
| Leonardo, Latino & white | I have like a little bump on my bicep on my arm. I've been told from inmates it's like a calcium buildup. It started off like a little marble, and it just got bigger. And they just said it's like calcium. But like when I do pushups and stuff, it kind of hurts. And it's gotten bigger, and probably like to the size of a quarter. And [medical] is like, oh, it's just calcium buildup. I'm just like, well, what are you going to let it build up 'til it's like a football? I don't get it...That was like a year ago. I'm going to probably [send a kite] when I go to D Unit and see what they say. But a lot of times that's what they do is, they wait 'til things are excessively bad. |
| Jacob, Native American | It takes a month to go through the seeing a doctor- once I see the doctor in like a week after I go to sick call then they examine it and I don’t know how long it takes for them to make the recommendation to have me sent down there but I do know from that day it takes a whole 30 days before I’ll actually see a doctor [specialist outside of prison] so we’re talking you know maybe two and a half months...Now our CIC board – if you ever leave the facility or you need certain medication or special types of treatment like it’ll cost you a few extra money there’s a board in Olympia that the facility provider’s supposed to call, explain their case and then if they approve it then you will go and do whatever it is they want you to do. But it takes a while for all that paperwork to go through and to get that meeting set up with Olympia until they do it. And once they make that decision 30 days from that day is when you go down to the hospital. |
| Emilio, Latino | I was stressed out, because the laceration was – it was big. It was like a – I thought it was infected, right? So I sent in the kite and it took them like a week and a half to do something. And the [medical] provider told me that, you know, she apologizes that – that she didn’t see me sooner. And due to my – all my [security] restrictions and conditions, she couldn’t see me. |

1. **Exacerbating Musculoskeletal Pain**

| **Name, race/ethnicity** | **Quotation** |
| --- | --- |
| Luis, Latino | [T]he pain I feel in my ribs. It’s screwed with me psychologically as well because I don’t know what it is…That’s the only thing that’s had me worried, it makes me sad, but anyway I say alright I can’t do anything about it myself. If something’s going to happen, it’ll happen. |
| Tim, white | I can't even do a pushup without causing unbelievable pain in my arms and in my shoulders, too…And my hands when I wake up in the morning - you ever seen a dead body? They go through stages of rigor mortis or whatever. In the morning time I wake up, my hands are completely locked, like literally frozen. |
| Isaac, Black & Latino | So I was talking to my guy [name redacted] and I was telling him like, "Man, you be having like quad or hamstring problems, man, you be having like pains and aches?" He's like, "Yeah, man. You know, I don't know what it is." It only happens when I come to IMU and I think maybe it's because you know, probably because we do a lot of laying down…[B]ecause I'd be thinking like oh, I'm laying in bed too much. Maybe my muscles are started to rot, you know, eating on themselves. |
| Eric, white | I've had a couple of physicals. I asked about my shoulder, you know, I wanted to get my shoulder rebuilt because it's coming out, it hurts. I'm in chronic pain all the time. |
