# Supplementary material for: The body in isolation: The physical health impacts of incarceration in solitary confinement
Source: PLoS One. 2020 Oct 9;15(10):e0238510. doi: 10.1371/journal.pone.0238510 (PMC7546459; doi:10.1371/journal.pone.0238510)
Supplement: Attachment — Submitted filename: Response to Reviewers.docx [file pone.0238510.s005.docx]

June 6^th^, 2020

Dear Dr. Knittel,

Thank you for the opportunity to revise our article, and for soliciting such meticulous and constructive reviewers. As you’ll see below, we have attended to each reviewer’s concerns, as well as to the additional editorial comments that you have provided. We have sought to attend to all of the reviewer’s feedback and revisions as thoughtfully as possible, while also working to condense and tighten the article. We look forward to any feedback from you and/or reviewers and again would like to express our gratitude for being considered for this special issue.

Per your suggestion, we have also provided additional information about our methods, including attaching the completed COREQ checklist as well as copies of our interview and survey instruments along with our submission materials.

The extensive edits we have made to our article made keeping all the track changes in our marked-up article extremely unwieldy. This caused issues in opening up the document and made it difficult to revise the document further. Given the drastic differences between our previous and new submission, we thought it would reasonable to accept a good portion of the track changes, while keeping enough to demonstrate the amount of edits made. In light of this, please note there is not a perfect match between the marked-up copy and the unmarked version of our submission.

Per the request of PLOS in-house editors, we have provided additional information regarding our data availability. We have included our responses below and approve that they be used to update our data availability statement. As part of fulfilling these requests, we also created a supplemental document charting a selection of additional and expanded quotes upon which our thematic analysis is based.

Sincerely,

Justin D. Strong, Corresponding Author

Answers to In-house Editors’ Requests in CAPS:

1. Please include a legend for figure 1.

A LEGNED HAS BEEN PROVIDED BEGINNING ON LINE 570 OF THE REVISED VERSION

2. Please upload a copy of Figure 1 which you refer to in your text on page 25. Or if the figure is no longer to be included as part of the submission please remove all reference to it within the text.

WE HAVE UPLOADED A COPY OF FIGURE 1 ALONG WITH OUR SUBMISSION MATERIALS

3. Thank you for providing a data availability statement and for explaining the restrictions on sharing the data underlying your study.

We note you have indicated the data is owned by a third party and contains confidential information.

Before we proceed, please address the following points:

1) We note you have mentioned in your contract with the Washington
Department of Corrections the data “will remain the
property of DOC and will be returned to DOC or destroyed when the work for which the information was required has been completed."

Please clarify if after the data has been destroyed, the DOC will still retain a copy of the data underlying your study.

THE DOC WILL RETAIN COPIES OF THE ADMINISTRATIVE RECORDS USED IN THIS STUDY; WE HAVE PROMISED TO RETURN OR DESTROY OUR ORIGINAL COPIES, HOWEVER.

2) We also note you provide information on how to obtain a similar data file from the Washington Department of Corrections. Please clarify if the same data used in this study can be accessed by future researchers, or if only a similar data file can be accessed.

IN THEORY, THE ADMINISTRATIVE DATA FILE COULD BE ACCESSED AGAIN BY FUTURE RESEARCHERS. IT IS DESCRIPTIVE DATA ABOUT THE WHOLE WASHINGTON PRISON POPULATION AT A POINT IN TIME, AND THESE ARE HISTORICAL RECORDS WADOC MAINTAINS IN PERPETUITY.

3) Please note, in the interest of long term access to data we do not allow authors to be the sole point on contact for fielding data access requests. Therefore, please provide a non author point of contact (such as an ethics committee, or the third party) where future researchers who meet the criteria to access confidential data can request access to the data underlying your study.

RESEARCHERS WOULD NEED TO CONTACT THE WASHINGTON DEPARTMENT OF CORRECTIONS. HERE IS THE PROCESS AND RELEVANT CONTACTS: <https://www.doc.wa.gov/information/data/research.htm#requests>

4) We note you are unable to share interview transcripts, but can provide a supplemental file with a list of quotations coded within various categories of analysis discussed in the paper. You are welcome to upload this as supporting information file at this time. If there was any codes used in the analysis of the transcripts, please also provide this.

WE HAVE INCLUDED A DOCUMENT OF SUPPLEMENTAL QUOATIONS.

5) Lastly, please confirm the authors had no special access privileges others would not have to the data underlying your study.

WE CONFIRM THE AUTHORS HAVE NO SPECIAL ACCESS PRIVILEGES OTHERS WOULD NOT HAVE TO THE DATA UNDERLYING OUR STUDY, BEYOND PATIENT NEGOTIATIONS WITH THE WASHINGTON DEPARTMENT OF CORRECTIONS ABOUT EXACTLY WHAT DATA WOULD BE SHARED FOR WHAT PURPOSES.

Response to Reviews in purple font:

1.We recommend that authors use the COREQ checklist or other relevant checklists listed by

the Equator Network such as the SRQR to ensure complete reporting (<http://journals.plos.org/plosone/s/submission-guidelines#loc-qualitative-research>). Submitting a completed checklist with the manuscript helps to ensure that qualitative work is reported rigorously.

Thank for this suggestion. Along with revising our methods section so that it discusses the COREQ checklist more directly, we have added an additional checklist of all 32 COREQ items along with our submission materials. We understand that this checklist is for peer-review purposes and would not be included in the publication of the article, but we are not opposed to the COREQ checklist being published as supporting information.

2. The manuscript as written both contains extraneous information (as highlighted by reviewer #1) and is missing important parts of the methods (as identified by both reviewers). While there is not a particular word count to target, I would recommend a very detailed revision for style with the goal of a succinct but comprehensive description of the work that was conducted.

Thank you for this helpful suggestion. We have done a very close read through of the document, revising and condensing for length wherever possible. Much of this was already in line with the reviewers’ comments, but we have focused on writing all sections as succinctly as possible, and we have reduced the overall wordcount in the manuscript by more than 1000 words.

Journal Requirements:

When submitting your revision, we need you to address these additional requirements:

1. Please ensure that your manuscript meets PLOS ONE's style requirements, including those for file naming. The PLOS ONE style templates can be found at <http://www.plosone.org/attachments/PLOSOne_formatting_sample_main_body.pdf> and <http://www.plosone.org/attachments/PLOSOne_formatting_sample_title_authors_affiliations.pdf>

We have reviewed the formatting guidelines and have made necessary changes to the title page, listing of authors, and main body of the text.

2. Please include additional information regarding the survey or questionnaire used in the study and ensure that you have provided sufficient details that others could replicate the analyses. For instance, if you developed a questionnaire as part of this study and it is not under a copyright more restrictive than CC-BY, please include a copy, in both the original language and English, as Supporting Information. Moreover, please include more details on how the questionnaire was pre-tested, and whether it was validated.

We provide the survey and interview instruments we designed for this study now as supporting information with our article (S1 Text) and we have added additional details about the development of the interview instrument in response to question 3 below, in line with the COREQ guidelines.

3. For qualitative studies, PLOS ONE suggests  consulting the COREQ guidelines: <http://intqhc.oxfordjournals.org/content/19/6/349> to ensure that all relevant information is provided (in this case we would appreciate more information about:  if a pilot study was tested; if bias issues were considered ). Moreover, please provide the interview guide used.

We found this list very helpful in drafting the article originally and have returned to it as revised. We have added a few sentences in response to the specific queries about pilot studies and bias issues. In terms of bias issues, we describe the demographics of our interviewers and note how assumptions and biases were addressed through the interview training (see “Data Collection Instruments” sub-section in methods). In terms of pilot studies, we note that the paper survey we used served as a pilot study, note that many interview questions we used had been previously used on other instruments, and explain that the final instrument was revised slightly after being used for the first set of interviews at the smallest institution included in the study (see “Research Team Training” sub-section in methods). We have included the final interview guide (S2 Text) as a supplement to this article. Finally, we have drafted our responses to the 32-item COREQ checklist and have included that along with our supplemental materials.

4. In your Data Availability statement, you have not specified where the minimal data set underlying the results described in your manuscript can be found. PLOS defines a study's minimal data set as the underlying data used to reach the conclusions drawn in the manuscript and any additional data required to replicate the reported study findings in their entirety. All PLOS journals require that the minimal data set be made fully available. For more information about our data policy, please see <http://journals.plos.org/plosone/s/data-availability>.

Upon re-submitting your revised manuscript, please upload your study’s minimal underlying data set as either Supporting Information files or to a stable, public repository and include the relevant URLs, DOIs, or accession numbers within your revised cover letter. For a list of acceptable repositories, please see <http://journals.plos.org/plosone/s/data-availability#loc-recommended-repositories>. Any potentially identifying patient information must be fully anonymized.

Important: If there are ethical or legal restrictions to sharing your data publicly, please explain these restrictions in detail. Please see our guidelines for more information on what we consider unacceptable restrictions to publicly sharing data: <http://journals.plos.org/plosone/s/data-availability#loc-unacceptable-data-access-restrictions>. Note that it is not acceptable for the authors to be the sole named individuals responsible for ensuring data access.

We will update your Data Availability statement to reflect the information you provide in your cover letter.

The administrative data we analyze in this paper is drawn from a confidential data file, shared with the research team for the limited purpose of evaluating patterns of solitary confinement use in the Washington Department of Corrections. More specifically, the data was provided pursuant to a “Limited Data Share Agreement,” codified in Contract No. K11273 with the Washington Department of Corrections. The contract requires that the data be stored in a secure environment with “access limited to the least number of staff needed to complete the purpose of the agreement.” The contract further states that the data “will remain the property of DOC and will be returned to DOC or destroyed when the work for which the information was required has been completed” and specifies explicitly that the data file cannot be “redisclosed or duplicated.” The restrictiveness of this agreement reflects the sensitivity of the data shared, including detailed records of individual prisoner’s case histories, even sub-sections of which could be inadvertently used to identify people promised confidentiality. The data, therefore, cannot be shared publicly.

If any researchers wish to obtain a similar datafile from the Washington Department of Corrections, the authors of this paper would be happy to consult with those researchers about the request and the process for obtaining the data. Moreover, it is a longer-term goal of this project’s authors to work with the Washington Department of Corrections to make our data requests and analytic processes public, but the analysis is still ongoing, and so that negotiation process about what forms of aggregated or de-identified data might ultimately be shareable is a conversation that must take place at some future date upon completion of all agreed-upon analyses related to this underlying administrative data set.

The interview data we analyze in this paper comes from transcriptions of in-depth, highly personal, one-on-one interviews. In line with social science norms around qualitative interview transcripts, the need to protect the identity of subjects, and the restrictions of our Institutional Review Board protocol, we cannot share the full transcripts of these interviews publicly. However, we would be happy to provide a supplemental file with a list of quotations coded within various categories of analysis discussed in the paper (from which we were usually only able to provide one quotation of many possible examples), if this would be of interest to the editors.

5. Your ethics statement must appear in the Methods section of your manuscript. If your ethics statement is written in any section besides the Methods, please move it to the Methods section and delete it from any other section. Please also ensure that your ethics statement is included in your manuscript, as the ethics section of your online submission will not be published alongside your manuscript.

We have now included more specific information about our ethics statement in the “interviews” subsection of the methods section of the manuscript: describing the IRB that approved this study, specifying the identifying number for the approval; and indicating the forms of consent (oral and written) obtained.

6. Please upload a copy of Figure 1, to which you refer in your text on page 15. If the figure is no longer to be included as part of the submission please remove all reference to it within the text.

Figure 1 has been included as part of the supplemental materials.

7. Please include a caption for figure 1

Figure 1 has been captioned accordingly: S1 Figure. Racial and Ethnic Composition of IMU Sample, General Prison Population, and Washington State, 2017

**Reviewer #1:** Overall, the manuscript presents novel data on an important topic. However, the paper needs to be significantly restructured and substantially shortened. Specific overarching comments are below.

We would like to begin by thanking you for your very helpful feedback. We have paid careful attention to condensing our introduction, expanding on our methods, and reserving the broader analysis and takeaway of our findings for the discussion section.

The Introduction is much too long. Some areas that can be cut and/or condensed include:

We really appreciate these thoughtful and specific suggestions for condensing the introduction and have implemented all of the suggestions below, as described in detail following the specific points.

Page 3, the first sentence can be deleted—this is a wordy intro sentence that is more confusing than helpful re orienting readers to the topic of the manuscript.

We wholeheartedly agree that this sentence is much too wordy. So that the sentence orients readers to the topic of the manuscript, we have made the following edit: “The health implications of solitary confinement has received increasing attention in recent years”

Page 4, line 74—this paragraph can be deleted. While the information presented in this paragraph is largely accurate, the authors do not explicitly connect this information to solitary confinement and introduce multiple other topics that they do not sufficiently incorporate/integrate into the rest of the manuscript.

This point is very well taken. We have deleted this paragraph entirely and from it have edited and repurposed lines 86-87 to lines 96-98 of the revision version.

Pages 5-6 line 109—the paragraph beginning at line 109 is much too lengthy and needs to be condensed significantly.

We have revised this paragraph by condensing lines 110-114 and removing the extraneous information on legal change and health policy on lines 123-128. Paragraph beginning on line 107 of the revised version reflects these changes.

One concern throughout is the use of the term “prisoners”. Please consider using person-first language and changing prisoners to persons who are incarcerated or persons incarcerated in prisons. While this terminology is wordier, it reduces the stigma associated with the term prisoner.

Thank you for raising this very important point. We have gone through the paper and replaced “prisoners” with people-first language, such as people incarcerated, people in prison, and people living in solitary confinement.

Methods:

This section is much too lengthy and is very confusing.

Again, we really appreciate these thoughtful and specific suggestions for both clarifying and condensing the methods, and have implemented your specific suggestions as described below. Throughout, we have sought to clearly organize the methods to delineate the multiple sources of data we analyze in this study, as well as direct reference to the elements of the COREQ checklist, per the handling editor’s request.

The information presented in pages 8-9 can be reduced to a paragraph or two at most. The paragraph beginning at line 192 can be deleted—too much extraneous detail is presented.

We condensed all information presented on pages 8-9 to two paragraphs, and the paragraph beginning on line 192 of the original submission has been deleted. This condensing also helped us to focus on re-organizing our presentation to specify the four key dimensions of our data collection and analysis: 1. Surveying of all people in IMU in Spring of 2017; 2. Interviews of 106 participants in Summer 2017 and follow-up interviews of 80 of these participants in summer of 2018; 3. Review of medical files of all interviewed participants who consented. 4. Analysis of administrative data from WADOC. This can be found in the paragraph beginning on line 140 of the revised version.

Paragraph beginning on line 201—this paragraph needs to be condensed quite a bit—there is much too much detail, the methods could be succinctly summarized in about half the space.

We have revised the paragraph on line 201 of the original version accordingly to remove all extraneous information, while still describing the extensive clinical training our team members underwent in preparing for this study, an important point for us to explain transparently, since studies on solitary confinement conducted at similar scales as ours typically lack such standardized clinical training. Please see the paragraph beginning on line 184 of the revised version. We have also provided additional subheadings to make clear the different aspects relevant to our study design, such as sampling, research team training, and interview. Doing so has allowed us to write these sections much more succinctly.

The authors state that they did not provide participants with an incentive? Why not? This is concerning given the amount of time participants provided during interviews. It is certainly possible to provide monetary incentives to incarcerated persons through placing funds on their commissary account.

We appreciate this point and are all committed to thinking carefully and being transparent about the ethical decisions inherent in research with vulnerable populations. While providing monetary incentives for participants is ideal in many settings, it was neither a realistic option, nor would it have been a clearly ethical procedure, for our study. Realistically, being able to provide monetary incentives would have required various administrative clearances that the WADOC was not willing to allow for the study, especially for people in solitary confinement, who have limited access to commissary accounts and funds therein. Moreover, in prison contexts, compensation is complicated, and it is more complicated the more restrictive the context; any compensation, in fact, might improperly work as an undue influence, or worse, a coercion to participate in research, especially for prisoners who might not otherwise have access to any funds whatsoever even to meet basic needs like buying soap or making a phone call to a family member. In fact, our participants repeatedly told us that getting time out of their cell and talking with us served as a form of compensation. As was expressed by many of our participants, being able to talk to someone other than a CO or fellow prisoner was a significant benefit of participating in the study. In acknowledgement of this important concern, we added a brief sentence explaining this decision in the “interviews” sub-section of the methods, lines 203-205 of the revised version.

Note that, per the request of the editor and PLOS policy, we have also included a specific ethics statement about our research review process and details of consent.

Data Collection

The first sentence can be deleted (the authors state this is a mixed method study elsewhere).

This paragraph has been revised accordingly.

The description of the survey questions is insufficient. Of the 96 questions, the authors only provide a cursory description of what these questions entail.

Here, we have, first, clarified that we conducted both a paper survey and in-person interviews, and we describe both the substance and format of the questions in more precise detail, specifying exactly how many questions were closed-ended and quantitative and how many were open-ended and qualitative, as well as specifying the quantitative subset of these survey questions we include in the analysis in the current paper. We also explain, to further clarify and respond to the editors’ suggestion, how the survey served as a pilot-instrument for the in-person interviews. Finally, we have included both the survey and interview instrument as supporting materials to our article. Please see S1 Text and S2 Text.

Relatedly, and much more of a concern, there is very limited information about the qualitative nature of some of the questions asked as part of the survey. Were some of the questions included on the survey open ended such that they would allow a more in-depth response? There is very little mention of whether or not there was clear structure to the questions, or if they were semi-structured, how many open-ended questions were included within the survey and what types of content areas such questions were supposed to cover. Additionally, the authors provide no detail or rationale as to which questions were asked qualitatively and which questions were more traditional quantitative type survey questions. Therefore, it is very difficult to understand how the data were collected and to assess the appropriateness of the analyses.

Having clarified that we conducted both a paper survey and in-person interviews, we elaborate on the organization of the semi-structured interview questions, detailing exactly how many questions were closed-ended or quantitative, and how these related to the bulk of the open-ended, qualitative survey questions. Having thus elaborated on the exact structure of the qualitative interview instrument, we note that we focus in this paper on text from anywhere within these qualitative interviews relating to physical health, better setting up our explanation of the coding process. Please see S2. Text as mentioned above.

Data analysis and reporting:

The amount of coding is substantial and is hard to understand given that there is no information about the overall structure of the interview instrument (e.g. what proportion was quantitative, what proportion was qualitative). Also, there is no consistent framework that appears to have been used to analyze the data. Nor is there a thematic approach to organizing the data, as is consistent with most qualitative analytical frameworks. It is also confusing as to which questions were asked at follow up (and related which of these were quantitative and which were qualitative).

First, in providing a copy of the interview instrument for review, and a more thorough discussion of the kinds of questions asked and topics covered, we have provided context in the “data collection instruments” sub-section, which we hope makes the subsequent “data analysis” sub-section easier to follow. Second, we have revised our explanation of our coding process to re-focus on the grounded theory method we applied overall, and especially the intermediate focused coding yielding the results presented in this paper, including explaining how the codes relate to the interview instrument questions asked. Our coding was extensive, as our interviews were incredibly rich and substantive, but this paper focuses on one narrow subset of those larger themes and codes, and we have sought to focus in on these themes and codes, explaining how they were derived, in the “data analysis” sub-section.

Results:

The first paragraph needs to be cut (or parts could be integrated into methods).

We have cut the first paragraph of the results section entirely, per your suggestion. We jump right into our first category of results, the prevalence of somatic concerns. Note that we re-organized the subsequent results categories, as we explain further below, to both promote clarity in presentation and to better separate results from discussion.

There are multiple areas where the authors insert sentences which provide context to their findings—this should be included in the Discussion, not Results. Specific instances of this are: lines 335-336; 376-379; 407-417 (should be included as limitations within the Discussion section); 514-516; 571-576; 582-583; 606-608.

Thank you for this careful specification of lines where we included context to our results. We have identified each of these lines and incorporated them into our Discussion.

Table 1 is not needed, this information can be presented briefly in the narrative.

Table 1 has been deleted. We made slight edits to the narrative to ensure that we’ve described the key differences in the populations and statistical tests/calculations.

Line 376: Don’t need the test statistic, p-value is sufficient

We removed the reference to the test statistic (and retained the p value). Note that in the parenthesis with the p value, we listed the statistical test conducted.

Table 2 also is not needed. Given the relatively small cell sizes, the authors can brief provide an overview of the data presented in Table 2. As is, there is too much detail about very small numbers of individuals—this needs to be more concisely summarized.

We have cut out Table 2, and made revisions to the text to ensure that we are fully describing the findings over time and by housing location in the narrative.

Line 418: need to re-orient the reader to the 225 number—it is difficult to remember where this comes from without going back through the manuscript.

We made slight revisions to the first sentence on this paragraph to provide more context about the surveys we collected. We hope that, in combination with our methods revisions more clearly specifying each of our data sources, this is now more clear.

The Emerging Symptoms title is confusing. These refer to new physical symptoms participants experiences resultant from solitary so this should be made more explicit. Also, some of the quotes in this section are too lengthy (e.g. quote beginning on line 450). Please do not include interviewer questions in the quote, the authors can provide context for the response and then provide the direct participant quote (as is typical for qualitative data reporting).

Overall, this section is not very well organized. It includes a host of issues that are not necessarily connected with the overall theme of “emerging symptoms”. Much of this section needs to be much more focused, which may mean the authors need to prioritize specific quotes representing specific symptoms or illnesses they wish to highlight.

The following section of Persistent Symptoms is similar in that it is fairly unfocused and lacks organization. Quotes such as the one beginning on line 624 need to be significantly condensed.

We found this suggestion perhaps the most generative, and have worked to re-organize these sections around three new titles (integrating suggestions from Reviewer #2 as well) and condensing the quotes and examples. First, we significantly reduced all block quotes and have removed all interviewer questions. We have also removed specific quotes that we felt were redundant to more descriptive experiences shared by other participants. Second, we re-ordered these sections around three new categories that make the relationship between participant symptoms and the institutional mechanisms of their confinement much more central, building up to what we see as the central contribution of this piece, about the interrelationship between solitary confinement and physical health symptoms. In this way, we re-focus on the organizational and behavioral factors that impact physical health of people in solitary confinement.

Discussion:

While the authors do document disproportionate numbers of people of color in their sample, the sample is relatively small so results should be interpreted with caution since they may not be representative.

We address the generalizability limitations of the study in our discussion section now, although we note that the disproportionalities we describe are significant, have been replicated in other studies of other systems, and exist even in a state with fewer people of color in the state population and in the prison system overall.

**Reviewer #2:** Thank you for the opportunity to review this manuscript on an issue that is important and particularly timely in view of an ongoing public health crisis that has extended into jails and prisons and imposed severe strains on housing and healthcare services in these settings. I believe this will be an important contribution to the literature if the authors can address a few concerns outlined below.

We would just like to begin by thanking you for the thoughtful feedback and suggestions where we may have a more robust discussion of our findings. In our discussion section we have now integrated the research and findings suggested below along with incorporating other empirical studies that add further support to our qualitative findings.

(1) When the authors, in their introduction, describe the work of some researchers who “have argued that the psychological harms of solitary confinement are limited or unverified”, they undersell the extent to which these studies are methodologically flawed and widely regarded as discredited. A more forceful summation of this limited literature may be helpful for readers who are not steeped in the relevant back-and-forth among experts in the field.

We appreciate this point and have added additional specifications of the exact problems with these studies in the subsequent sentence.

(2) On pg 23, P1 the authors describe “the most resilient prisoners” but don’t offer a definition of resilience and it is not clear what is meant here. Are authors referring to the specific and widely used medical definition of the term? If so, how have researchers identified “resilient” patients? A non-medical use of resilient here may also be worth reconsidering for at least two reasons. First, defenders of solitary confinement have pointed to the “resilience” of some to justify the practice’s widespread use. And second, referring to someone as resilient under such harsh conditions may require a more thorough longitudinal assessment of mental health and well-being than was available in this study.

Thank you for prompting us with this very thoughtful concern. Our use of resilience was largely colloquial and have changed the phrasing to better emphasize our finding that people in solitary confinement utilize exercise as a way to manage their physical and emotional stress. Here we write on lines 410-412 of the revised version, “As Kai suggests, in the IMU, exercise functions not only as a means to practice physical fitness, but also to provide structure for people to manage their day, as well as the mental strain of being in isolation.”

(3) Regarding Roland’s experience: data from the Ashker litigation (Hawkley’s expert testimony) suggests a heightened risk of hypertension associated with long-term isolation even in a relatively young population. This may provide important context for this finding – and raises the question of whether solitary confinement has contributed to a rapid rise in the number of deaths among prisoners attributable to cardiovascular disease – as well as the high relative risk of mortality due to cardiovascular disease among recently released prisoners.

We really appreciate this connection being drawn to our work. We were excited to build-off of Williams et al. (2019) study on cardiovascular disease (cited in our literature review) and which we now cite, along with Hawkley’s testimony, in the discussion section related to the policy deprivations Roland elucidates. The turn towards understanding the physical health effect of solitary confinement is extremely encouraging and Hawkely and Willaims et al. provide incredibly strong empirical evidence of the association between hypertension and long-term isolation. Indeed, we hope that we may continue to contribute to such an important research focus in further studies. Accurately describing the pain and suffering of solitary confinement is ultimately an ethical commitment to those incarcerated and extends to building public knowledge of what transpires in the name of safety and security.

(4) Roland’s case also raises the unique patient-provider trust / dual loyalty issues present in U.S. jails and prisons. Correctional healthcare clinicians commonly complain about attention- and drug-seeking behavior among residents, but hiding symptoms for fear of housing changes etc may be the more prevalent behavior (and certainly the more worrying). Solitary confinement likely exacerbates this mistrust – especially since some systems ask clinicians to assess patient suitability for isolation (see recently resolved Johnson v. PA DOC litigation). This is just another example of a mechanism by which solitary confinement likely leads to physical health harm and one that the authors may wish to consider raising in view of both Roland’s and Blake’s powerful and profoundly upsetting testimony.

We absolutely agree with this point and have connected dual loyalty to Roland and Blake’s experiences, as well as provide a broader connection to dual loyalty and healthcare issues in solitary confinement, in our discussion, lines, 686-689 of the revised version. We hope to build upon this topic more specifically in later works as it warrants an intentional and thorough examination of its own.

(5) Similarly, the compelling and important description of the way in which care is often administered in these units at bottom of pg 27 highlights another example of an important way in which solitary poses high and unnecessary risk of physical health harm. As the authors suggest, cell-front assessments are neither medically nor ethically appropriate nor are medication restrictions like those the authors describe. Julian’s and Carl’s cases – though in different ways – are also representative of this profoundly disturbing reality: that solitary confinement carries an additional punishment of substandard access to and quality of health care. This suggests that there is no safe version of solitary confinement – the only appropriate conclusion given the host of ways in which physical health is harmed by these conditions is to eliminate its practice for anything longer than hours-to-day, as recommended in the Istanbul Statement and required by the Mandela Rules (though 15 days specifically is arbitrary and likely overly long from both correctional efficacy and health perspectives).

We appreciate such a close-reading of our study. This is precisely why we have reworked the analysis of our findings to make more concrete the analysis of institutional mechanisms. We have adapted our language on lines 508-510 of the revised version to emphasize the point raised on access to health care: “As our participants’ experiences suggest, solitary confinement carries an additional punishment of substandard access to health care.”

(6) The musculoskeletal pain finding, symptomatic of likely musculoskeletal conditions that are almost certainly exacerbated in these conditions, may warrant its own treatment / subheading for both how common it was in the study and how it likely exists in both the emerging and persistent categories. Both methodologically and narratively, it is worth highlighting the finding that solitary confinement inflicts very real, distressing, and persistent physical pain on those subjected to it – consistent with many other historical torture schemes, none of which remains as widely used as this one.

Yes, we greatly appreciate this point and have devoted a sub-section of our qualitative findings to persistent pain. As we frame the discussion of this section, the inability to engage in effective pain management and to be an active participant in one’s own well-being is a serious health effect of isolation and is likely comorbid with other mental health symptoms. As we also frame in the discussion section, lines 612-613 of the revised version, musculoskeletal pain also poses the risk of disability.

(7) I point to some areas above where the authors might consider a more robust discussion or explicit statement of the myriad ways in which their evidence suggests solitary confinement undermines physical health because I believe they are points worth making in themselves, and are well-supported by the compelling data reported in this study. But I also raise these issues to support my main concern with the manuscript: the calls for future studies and future research that animate much of the paper’s discussion section. The authors correctly cite a wealth of evidence – and an international consensus of experts from a number of disciplines – attesting to the mental health harms of solitary confinement. They also rightly point to a relative dearth of knowledge – despite compelling anecdotal evidence from case studies and lawsuits – describing the physical health harms of solitary confinement. They then present exceedingly compelling evidence that solitary confinement is associated with multiple and varied physical health harms with likely multiple and varied etiologies, many of which very clearly arise from or at the very least are exacerbated by this form of housing (e.g. health harms arising from inadequate access to care that is more readily available in general population, health harms arising from behavioral changes necessitated by isolation, health harms arising from stress that residents attribute to their housing status, and others). Yet they conclude: “physical suffering reveals itself to be a crucial dimension of experience in solitary confinement that must be explored in greater depth and detail.” Must it?

The authors are wise to call for a greater understanding of the pains of imprisonment – and I agree that this study provides evidence of a clear need for additional research into the health effects of incarceration – a practice that though it may be significantly reduced in the decades to come, will continue to directly effect hundreds of thousands of Americans for the foreseeable future. But there is no compelling correctional, public safety, criminological, or certainly public health rationale for the use of solitary confinement beyond hours or days (nor do the authors attempt to cite one). The residents in this study had an average stay of 14.5 months in these conditions. Thanks in great measure to the authors’ work, it seems the only reasonable conclusion to draw is that, based on a wealth of scientific evidence and an overwhelming international consensus among legal, health, and human rights experts, the practice should be immediately ended.

To suggest that additional research is needed on the subject risks that readers, including policymakers and correctional professionals, could misinterpret the authors’ call as implicit acceptance of a practice that, based on the authors own evidence, amounts to torture. This concern need not necessitate a major revision of the discussion. Rather, every piece of evidence and context that the authors marshal in favor of further research could easily – and more accurately – be presented in support of a call to consider the science well past settled on this issue and to end this practice immediately.

We found these suggestions incredibly helpful and fruitful in thinking through how to frame our discussion and the implications of this work. We have re-framed the discussion (also incorporating reviewer #1s suggestions that we move much of the contextualization in the findings sections into the discussion as well) to focus on: 1. The physical health symptoms we document are potentially quite dangerous, show how punishment itself is experienced in health-related way, and likely disparately impact prisoners of color. 2. To the extent there are limitations to our work, they suggest we are undercounting any harms we identify; 3. Specifying physical problems in solitary confinement elucidates the severities of the conditions and suggests physical suffering is one of these severities; 4. Physical symptoms in solitary confinement provide a framework for talking about the pains of imprisonment more generally, and especially the co-morbidity of physical and mental suffering; 5. Physical symptoms in solitary confinement magnify mechanisms exacerbating health problems in prison, especially the problem of dual loyalty; 6. Understanding the health problems of those in solitary is a public health problem, because so many people both experience solitary and return to our communities. We need to identify the problems so we can mitigate them. In sum, with this re-framing, we state clearly that the harms of solitary are clear, but understanding them is important for thinking more broadly about the harms of incarceration more generally, which seems much less likely than solitary to be abandoned anytime soon.

(8) Related to this concern is my recommendation that the authors consider foregrounding their primary research question and corresponding results: that people in solitary confinement report multiple and varied physical health harms, with likely multiple and varied etiologies, including persistent physical pain that very likely either arises from or is exacerbated by the physical conditions that solitary confinement imposes. In order to foreground these critical findings, the authors should consider re-ordering their findings and discussion of racial disparities. As currently drafted – both in the introduction and, to a lesser extent, in the discussion – the paper is sometimes presented as primarily a disparities study. Certainly, there is an important sub-analysis in this work that speaks to the deep and longstanding injustice visited upon people of color at every intersection of the criminal justice system, an injustice that is first serialized then magnified in institutions of corrections like nowhere else. But as I suggest above, the paper’s main findings make such compelling medical, ethical, and moral cases for the elimination of long-term solitary confinement for all people regardless of race. These findings should be featured accordingly.

Again, thank you for such helpful and generous feedback. We have changed the ordering of our findings to discuss our qualitative interviews first, which then broaches a broader discussion of the disparate placement of people of color in solitary confinement based on our administrative data analysis. We agree wholeheartedly that this makes for a far more nuanced intervention in health inequity research. Not only are people in solitary confinement subject to an intensification of health inequities that people in prison face in general, but this is further complicated by the racial disparities of incarceration and placement in solitary confinement.

(9) Mentally ill and transgender residents are also disproportionately likely to be, first, incarcerated and, second, placed in solitary confinement – two important disparities that may also give power to the paper’s main findings.

This is an excellent point. We have added a sentence in our discussion about how the disparate impact on minority populations we document likely also applies to these other vulnerable populations disproportionately likely to be placed in solitary confinement.

(10) Finally, the authors should address if they believe that their findings are generalizable to other correctional systems in the U.S. They lay a foundation - when they describe WA DOC as progressive and reform-oriented - to suggest that their findings if anything underrepresent the likely harm to health being done by this practice day in and day out nationwide. But if they agree with this interpretation of their framing, they should make a more explicit case for generalizability.

We appreciate this suggestion. We have added a paragraph to the discussion in which we discuss the limitations of this study in terms of precise generalizability, but note that all of the limitations speak to a likely under-representation of harms at best.
