# Supplementary material for: The body in isolation: The physical health impacts of incarceration in solitary confinement
Source: PLoS One. 2020 Oct 9;15(10):e0238510. doi: 10.1371/journal.pone.0238510 (PMC7546459; doi:10.1371/journal.pone.0238510)
Supplement: Attachment — Submitted filename: Response to Reviewers.docx [file pone.0238510.s006.docx]

July 27, 2020

Dear Dr. Knittel,

Thank you for granting us another opportunity to revise our article. As you’ll see below, we have attended to each of the requested edits. Once again, we think our article is much stronger thanks to your and the reviewers’ comments, and we are encouraged by the prospect of acceptance. It has been a rewarding experience working with *PLOS ONE*.

We look forward to anymore feedback and would like to express again our gratitude for being considered for this special issue.

Sincerely,

Justin D. Strong, Corresponding Author

Response in purple font:

1. Line 141-144: The sample sizes are included elsewhere, so please remove them here and just list the types of data that were used.

Thank you, this revision has been made accordingly.

2. Line 145: Discussion of the robustness of the methodology compared with other studies belongs in the discussion section, not the methods. This can be removed or moved to the discussion section.

We have moved this point on the robustness of our methodology to the discussion section, which is now on lines 612-613 of the manuscript copy.

3. Participant Sampling: The response rates should be in the results section. Comparison to other studies may be included there, although that is best moved to the discussion section.

We have moved the description of our response rates to the results section, along with the brief comparison to other studies, so as to provide context for the robustness of our results. Please see lines 295-299 of the manuscript.

4. Line 167: Any description of the sample should be in the results, not the methods. Readers will expect a brief summary of the demographics at that point, rather than just a reference to another paper.

Thank you for this feedback. We have made this edit accordingly now providing a more explicit description of our sample, including a reference to the racial demographics of the sample at the beginning of the results section. Please see lines 299-307 of the manuscript.

5. Line 177: Was the review of medical records done only for individuals re-interviewed in 2018, as suggested by including this in the paragraph describing the re-interviews, or for all interviewees? Please review this section one more time for clarity.

Thank you for noting this point of confusion. Medical files were reviewed with the initial round of interviews in 2017. This detail has been revised for clarity. (Please also note that we added in a few additional references in the text to medical files reviewed, to better utilize this data source.)

6. Line 619: The authors seem to gloss over the difference in disparities between incarceration overall and solitary confinement. Consider reworking this paragraph slightly to acknowledge that not all people of color seem to experience solitary confinement disproportionately in WADOC, but that even though the disparities may be less striking than when looking at incarceration overall, disparities remain and the effects may be more substantial.

We appreciate this point and have added a bit more nuance to our explanation beginning on line 629 or the manuscript. Here we acknowledge how different races have different prevalances of solitary confinement experiences.

7. Line 631: I don’t understand what the phrase “and reproductive health” means in terms of risk of incarceration.

Indeed, our phrasing was confusing. We have clarified and simplified this point by just mentioning the specific example of “pregnant women,” a population of central interest in the study cited. Please see line 640 of the manuscript.

8. Please review the references for completeness.

In addition to reviewing the references for completeness, we read through the document carefully to check for any typos or other confusing phrasings. We also inserted two small clarifications about DOC policies, following up on conversations with research staff within the Department.
